# Comprehensive Two-Dimensional Gas Chromatography–Mass Spectrometry as a Tool for the Untargeted Study of Hop and Their Metabolites

**DOI:** 10.3390/metabo14040237

**Published:** 2024-04-19

**Authors:** Glaucimar A. P. Resende, Michelle S. S. Amaral, Bruno G. Botelho, Philip J. Marriott

**Affiliations:** 1 Australian Centre for Research on Separation Science, School of Chemistry, Monash University, Wellington Road, Clayton, VIC 3800, Australia; glaucimarresende@ufmg.br (G.A.P.R.); michelle.amaral@monash.edu (M.S.S.A.); 2 Chemistry Department, Universidade Federal de Minas Gerais, Belo Horizonte 31270-901, Brazil; botelhobg@ufmg.br

**Keywords:** headspace-SPME, GC×GC–MS, *Humulus lupulus*, essential oil, secondary metabolites

## Abstract

Since hop secondary metabolites have a direct correlation with the quality of beer and other hop-based beverages, and the volatile fraction of hop has a complex composition, requiring effective separation, here we explore the application of headspace solid-phase microextraction as a sample preparation method, coupled with comprehensive two-dimensional gas chromatography–mass spectrometry (GC×GC–MS) analysis. The methodology involved the use of a DVB/PDMS fibre with 500 mg of hop cone powder, extracted for 40 min at 50 °C, for both GC–MS and GC×GC–MS. The varieties Azacca, Cascade, Enigma, Loral, and Zappa were studied comprehensively. The results demonstrate that GC×GC–MS increases the number of peaks by over 300% compared to classical GC–MS. Overall, 137 compounds were identified or tentatively identified and categorised into 10 classes, representing between 87.6% and 96.9% of the total peak area. The composition revealed the highest concentration of sesquiterpene hydrocarbons for Enigma, whilst Zappa showed a relatively significant concentration of monoterpene hydrocarbons. Principal component analysis for all compounds and classes, along with hierarchical cluster analysis, indicated similarities between Zappa and Cascade, and Azacca and Loral. In conclusion, this method presents an optimistic advancement in hop metabolite studies with a simple and established sample preparation procedure in combination with an effective separation technique.

## 1. Introduction

Hop (*Humulus lupulus* L.) is a dioecious and diploid plant from the family Cannabaceae and is an extremely important raw material in drinks and beverages, such as sparkling teas and soft drinks and with major use in brewing. The use of hop as an ingredient was a significant turning point in the history of beer, increasing the beverage’s popularity and changing the physical/chemical properties of the beverage because of the secondary metabolites present in hop [1,2]. Nowadays, hop is the most expensive ingredient in brewing, with a market valued at USD 7.8 billion in 2022 and a growth prospect of more than USD 5.5 billion by 2030 [3]. Hop flavour is associated with the female flowers or strobili (termed cones) having a yellow powder named lupulin, which is the major source of hop aroma and is composed of acids, essential oils, and resins. Hop improves the aroma, stability properties, and bittering characteristics of the final product [2,4]. 

The composition of hop is complex and has an intrinsic characteristic based on properties such as variety/cultivar, geographic origin, environmental factors, and stability, among others [4]. The flavour, odour, and other physicochemical properties of hop are generally associated with five key categories of components: alpha acids, beta acids, polyphenols, terpenoids, and thiols. Alpha acids are responsible for the sensation of bitterness and foam stability, representing a mass fraction of 2 to 20% of the hop composition [5,6]. Beta acids influence the aromatic profile of the beer and are present in lower concentrations, due to their biochemical transformation during processing of the beverage. Polyphenols, such as tannins and flavonoids, act as stabilising agents in beer, influencing turbidity and flavour [6]. Finally, hydrocarbon and oxygenated terpenoids, as well as thiols, are some of the classes of components found in hop essential oil, which reportedly include more than 300 aroma compounds [4,7]. Essential oil compounds undergo biotransformation by yeast fermentation, such as geraniol generating beta-citronellol and, finally, the esterification product citronellyl acetate [8].

Numerous hop varieties are currently available in the market, with more than 100 cultivars being patented in the last decade and new products being continually developed and reported [9]. There are 363 results for *Humulus lupulus* species–metabolite relationships in the KNApSAcK Core System database [10], which aids in searching metabolomics and analytical studies. Metabolomics is a defined research field that has developed over the last 25 years, which involves the comprehensive analysis of small molecules (metabolites) present in complex biological matrices. Within this broad field, there are a number of different sub-disciplines that study the behaviour of matrices such as hop proteomics [11], genomics [12], beeromics [13], hopomics [14], and volatilomics [15].

Regarding the analysis of volatile organic compounds (VOCs) and volatilomics, gas chromatography (GC) is a standardised technique that has been applied to the determination of plant metabolites for more than 50 years [16]. Multiple analytical studies have targeted key aroma compounds in hop essential oil, such as myrcene (related to fresh odour), and linalool and geraniol (both related to floral notes) [17]. GC has also been applied in numerous analytical approaches for hop studies, such as chemical profiling [18,19,20], comparison of different types of sample preparation [21,22,23], and the study of the effect of hop in the brewing process [24,25].

Sun et al. [26] reviewed research associated with different sample preparation methods, such as steam distillation [17,27], simultaneous distillation extraction [25], dynamic [18] and static [19] headspace, direct thermal desorption [21], direct microwave desorption [28], liquid–liquid extraction (LLE) [29], stir bar sorptive extraction (SBSE) [23], and headspace solid-phase microextraction (HS-SPME) applied to hop [22,30]. This last method attracts attention as being a “greener” approach since it is a solventless technique using sorptive polymeric fibres, is simple, versatile, and has a wide applications base, with SPME reported for samples in the environmental industry, food, drugs, and other areas, although the quantitative application of SPME is rather complex [22,31].

According to the level of complexity of the hop matrix, the full resolution and identification of compounds can be difficult to achieve. Comprehensive two-dimensional gas chromatography (GC×GC) is designed for such multi-component samples [22]. Advantages brought by GC×GC compared with conventional GC include enhancement of separation power, increased peak capacity, and increase in sensitivity [32].

The use of GC×GC in the metabolomics field with respect to performance attributes was shown by Wong and coworkers [33]. In that study, GC×GC coupled with high-resolution quadrupole time-of-flight mass spectrometry (qTOFMS) was applied to samples of Eucalyptus spp. leaf oils, characterising four different species and their untargeted metabolic profiles [33]. The expression of metabolomic profiles was also extended to hop for the study of new genotypes, chemotyped by Yan and coworkers [34]. 

The application of GC×GC to hop analysis (Table 1) has been reported by thirteen research studies between 2003 and 2019, according to the Web of Science database, and data are summarised here as illustrative of capabilities for untargeted metabolomics. The first study was published 20 years ago and shows the potential of the technique for the analysis of hop essential oil [35]. With the development of the GC×GC, different setups were described, such as the parallel comprehensive two-dimensional gas chromatography (2GC×2GC) [36], and a range of detectors and modulators. Greater resolution power was achieved more recently, with the largest number of compounds reported by Yan and collaborators [34], who separated 306 and identified 99 compounds in their samples. Table 1 shows sample preparation and the column set applied in each study. Not only were comprehensive analyses of hop presented, but also the detection of compounds such as 4-mercapto-4-methylpentan-2-one (4MMP) (IUPAC: 4-methyl-4-sulfanylpentan-2-one (4MSP)). 4MSP is a black-currant-like odorant with an odour detection threshold as low as 0.00055 µg L^−1^ in beers [37], and these studies explore the advantage of the improved sensitivity of the GC×GC system. Odorants in hop samples were also reported in other studies such as that of Eyres, Marriott, and Dufour [38], which were concerned with the detection and possible identification of the aroma-active compounds/regions present in the samples. 

The aim of the present study was the analysis of different commercial hop samples, applying HS-SPME and GC×GC techniques to obtain the chemical profile and chemotyping of five different hop varieties, to highlight the general value of GC×GC to generate valuable information for characterisation and metabolomics studies. Thus, the primary objective was to demonstrate the applicability of this new methodology for volatile metabolite profiling of hops and associated benefits. From the accompanying literature review (Table 1), we observed that comprehensive analyses combining SPME and GC×GC techniques for hop analysis were not previously reported in the literature. This paper presents the first report of HS-SPME-GC×GC–MS for hop samples, establishing its capacity and presenting new data on the volatile profile of different species of hop.

## 2. Materials and Methods

### 2.1. Chemicals and Samples

The chemical compounds and standards used in this work were hexane (HPLC grade), ethyl hexanoate (98%), and methanol (HPLC grade) from Merck (Darmstadt, Germany); 2-octanol (99%), undecane (99%), (+) camphene (80%), β-myrcene (90%), D-limonene (97%), linalool (95%), geraniol (98%), geranyl acetate (97%), humulene (96%), (−)-caryophyllene oxide (97%), and n-alkanes C_8_–C_21_ (99%) provided by Sigma Aldrich (Castle Hill, NSW, Australia); and α-pinene (95%), β-pinene (94%), and caryophyllene (90%) from TCI (Tokyo, Japan). 

The hop samples Azacca (AZAC) and Loral (LORA) were kindly donated by Carlton & United Breweries (Asahi-CUB, Abbotsford, Australia) and the samples Cascade (CASC), Enigma (ENIG), and Zappa (ZAPP) were obtained from various other local suppliers. All samples were obtained in the format of pellet type 90, stored in the presence of nitrogen, and kept in a freezer until ready for analysis. More information about the hop samples is presented in Table S1 of the Supplementary Materials. 

### 2.2. Sample Preparation 

**Figure 1:**
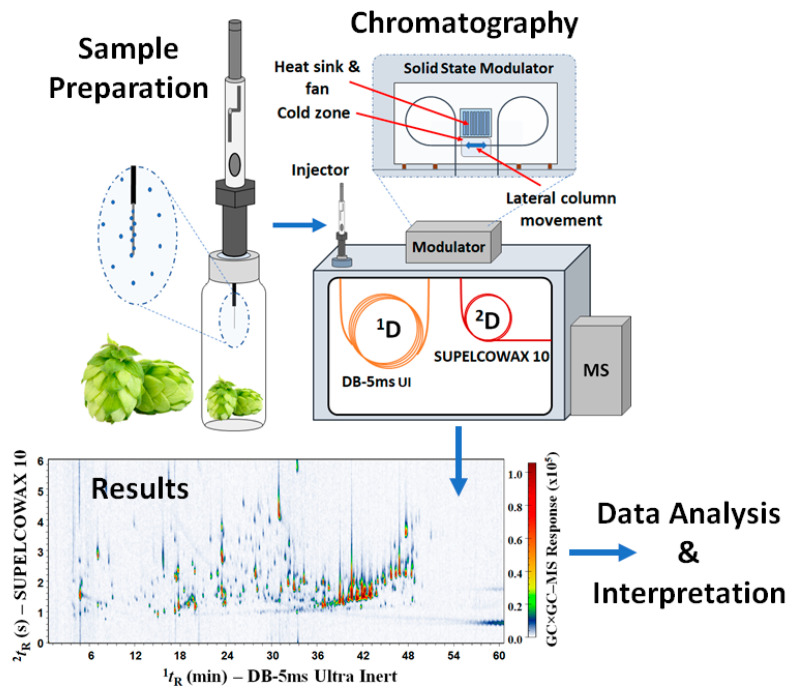
Schematic representation of the HS–SPME and GC×GC–MS analyses of hop.

The sample preparation and analysis procedure (Figure 1) was adapted and optimised based on previous studies [20,22,46]. The data regarding this initial examination of various experimental parameters, including the choice of the SPME fibres, among 65 µm DVB/PDMS (pink), 50/30 µm DVB/CAR/PDMS (grey), 100 µm PDMS (red), and 85 µm CAR/PDMS (blue), will be reported elsewhere. The following conditions were selected for the present study. HS-SPME was performed with a manual holder and a 65 µm DVB/PDMS (pink) fibre from Supelco (Castle Hill, Australia), conditioned in accordance with the manufacturer’s guidelines. In the first step, the pellets were ground in a mortar and pestle with 500 mg taken into a 20 mL screw top vial with clear glass. The vials were heated at 50 °C using a hot plate, with an equilibrium time of 10 min and 30 min for sorption. After extraction, the DVB/PDMS (pink) fibre was introduced into the GC injector and desorbed for 3 min. 

The retention index (R.I.) was calculated by the van den Dool and Kratz equation (Equation (1)) with a series of alkanes (C_8_–C_21_) analysed using 20 µL of 100 mg L^−1^ C_8_–C_15_ in hexane, 5 µL of 100 mg L^−1^ C_16_–C_21_ in hexane, and extracted by HS-SPME (same conditions as for the samples). The aliquots were introduced directly into a 20 mL clear glass vial with a screw top for extraction as above.

(1) $$I_{x}=100n+100\frac{\left(t_{Rx}-t_{Rn}\right)}{\left(t_{Rn+1}-t_{Rn}\right)}$$

where “*I_x_*” is the retention index, “*n*” is the number of carbons in the alkane prior to the analyte being determined, “*t*_Rx_” is the retention time of the analyte, “*t*_Rn_” is the retention time of the alkane prior to the analyte, and “*t*_Rn+1_” is the retention time of the alkane after the analyte.

### 2.3. Gas Chromatography (GC–MS and GC×GC–MS)

Chromatographic analyses were performed using two different systems. The GC–MS system was an Agilent 7890A GC with a 7000 triple quadrupole MS (Agilent Technologies, Mulgrave, Australia), with the first quadrupole operated with total ion transfer. The GC×GC–MS system was an Agilent 7890A GC with a 5975C single quadrupole MS (Agilent Technologies) and included an SSM1800 solid-state modulator (SSM) system from J&X (J&X Technologies, Nanjing, China).

The GC–MS method was adapted from hop studies in the literature [22] and included a DB-5ms UI column (30 m × 0.25 mm I.D. × 0.25 µm *d*_f_) connected to the MS transfer line by a deactivated fused silica (DFS, 1.0 m × 0.18 mm I.D.) and a glass press fit. Helium, grade 99.99%, was used as a carrier gas in constant flow (1.0 mL min^−1^). The injector was set in split mode (50:1) at 250 °C. The oven program was 50 °C (3 min), 3 °C min^−1^ to 200 °C, and 10 °C min^−1^ to 240 °C (3 min) (60 min analysis time). The MS settings were as follows: transfer line 240 °C, source 250 °C, quadrupole 150 °C, electron ionisation energy 70 eV, scan mode 35–350 *m*/*z*, and scan time of 300 ms.

The GC×GC–MS method including the solid-state modulator operation was also based on previous research [40,47] and included a DB-5ms UI ^1^D column (30 m × 0.25 mm I.D. × 0.25 µm *d*_f_) and a SUPELCOWAX 10 ^2^D column (1.0 m × 0.10 mm I.D. × 0.10 µm *d*_f_). Deactivated fused silica capillaries were used as the modulator column (1.0 m × 0.15 mm I.D.) and the transfer line (0.43 m × 0.10 mm I.D.), and were connected to the main columns using glass press fit connectors. The GC temperature programme was the same as used in GC–MS analyses, except for the MS source 230 °C, quadrupole 150 °C, threshold 80, 12500 scans (u/s), and 22.8 scan/s. The solid-state modulator configuration was adapted from the literature [47] and included the same temperature program of the GC oven for the modulator entry oven and a 20 °C offset above the GC oven for the exit oven. The trap temperature program was −50 °C (8 min) and 2 °C min^−1^ to −20 °C (37 min). 

### 2.4. Data Analysis 

The GC–MS and GC×GC–MS data processing and chromatographic analyses were performed using Agilent MassHunter workstation Qualitative Analysis version 10.0 (Agilent Technologies, Santa Clara, CA, USA) and J&X Canvas W1.5.14.30115 (J&X Technologies, Shanghai, China) software. The MS identification was performed using the NIST 11 mass spectrometry library. 

Statistical analysis was performed by Excel^®^ (Microsoft, Redmond, WA, USA), MATLAB^®^ software, version 7.13 (MathWorks, Natick, MA, USA), including the PLS Toolbox, version 6.5 (Eigenvector Technologies, Manson, WA, USA). The multivariate statistics analysis included principal component analysis (PCA) and hierarchical cluster analysis (HCA). This analysis included pre-processing autoscaling and removal of outliers in the graphs and Hotelling’s T2 versus Q residuals. Two types of PCA were performed, viz. by the areas (%) of compounds found in the samples, and by the sum of areas (%) of the classes of compounds. For HCA for the classes of compounds, Ward’s method was applied and PCA with 3 components. 

## 3. Results and Discussion

**Figure 2:**
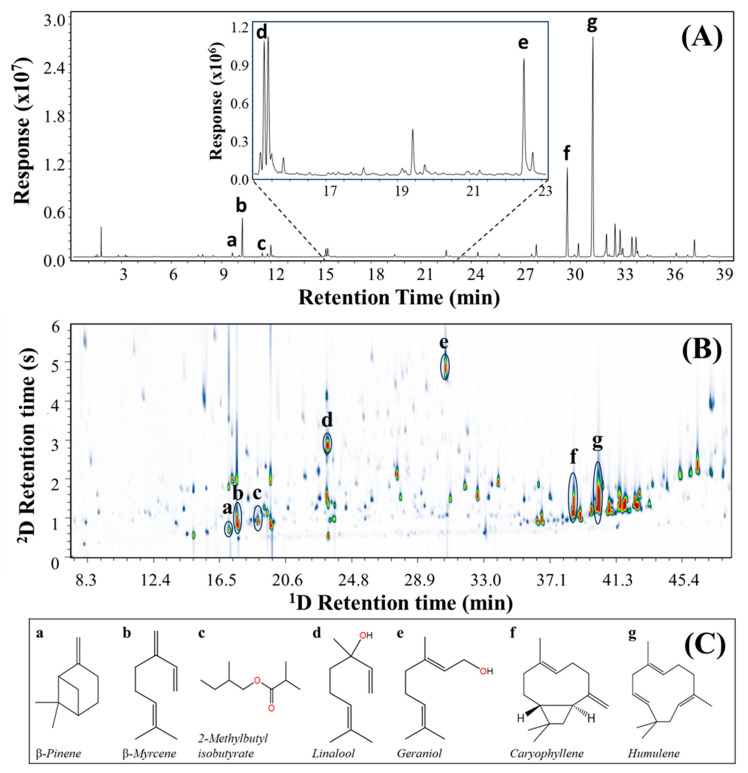
A comparison between two chromatograms of (**A**) GC–MS and (**B**) GC×GC–MS applying HS–SPME for Cascade hop, where the GC–MS and ^1^D column of GC×GC-MS is a DB-5ms UI (30 m × 0.25 mm I.D. × 0.25 µm *d*_f_), the ^2^D column is SUPELCOWAX 10 (1.0 m × 0.10 mm I.D. × 0.10 µm *d*_f_), and the structure of the selected compounds (**C**) is represented by a–g indicated in (**A**,**B**).

One of the crucial steps in the analysis of hop varieties and their volatile compounds is to achieve an acceptable separation of peaks, leading to the increase in selectivity and improvement in the identification of substances due to reduced overlapping interferences. The complexity of hop samples can be represented by the single separation dimension for the Cascade hop GC–MS result shown in Figure 2A, for which the given GC column is apparently of conventional separation quality with a peak capacity for the 32 min analysis of approximately 300; this indicates responses that have considerable peak overlap. For example, “d” (15.4 min) is a linalool with a cluster of poorly resolved peaks, and peak “e” at 22.0 min (geraniol) has an evident shoulder before a peak on the geraniol tail. The molecular composition of such regions will be indeterminate. The complexity becomes immediately clear when GC–MS (Figure 2A) is compared with GC×GC–MS (Figure 2B) with retention times on the DB-5ms UI columns arranged to show the same peaks aligned vertically in each panel. The existence of significantly more compounds is confirmed in the latter GC×GC result, simply through the addition of the second dimension (^2^D) separation. Being a more polar column (wax-type), the ^2^D retention proceeds from less to more polar along this axis, and so this indicates the relative polarity of compounds that coeluted on the ^1^D column, and can serve as a check for possible chemical class. This leads to the notion of molecular structure–retention relationships in GC×GC [48]. It is evident that the 2D presentation of GC×GC with most peaks resolved covers a large proportion of the volatile (headspace) composition of the sample, leading to the suggestion that this embodies the requirements for comprehensive metabolite profiling [49] and is a quintessential untargeted method for volatile compounds [50].

The GC×GC–MS configuration scenario for metabolite analysis, generating a greater number of peaks, should also include better identification by the MS through more intense peaks that improve minor constituent detectability, fewer matrix interferences, and less phase bleeding. Here GC–MS analysis was similar to that in the literature [22]. For GC×GC–MS, ^1^D was a nonpolar DB-5ms UI phase column (5% phenyl methylpolysiloxane), and two ^2^D phase columns were tested: an intermediate polarity BPX50 phase (50% phenyl polysilphenylene-siloxane) and a polar phase (SUPELCOWAX 10; polyethylene glycol), both of the same dimensions. The results (Figure S1) displayed an enhanced separation with the polar ^2^D phase, better resolution of peaks, and a larger number of recorded metabolites. The modulation period (*P*_M_) used for GC×GC corresponds to the time available for completion of the ^2^D analysis before the wrap-around might occur. Times of 4, 5, 6, and 7 s were tested, with the best separation for 6 s, and a little wrap-around. As for the choice of the SPME fibre, the higher number of peaks, which we correlate with better coverage of total metabolites, was the pink fibre (Figure S2).

The SPME method presented in this work is advantageous for the study of volatiles in hop. Compared to other methods previously applied (Table 1), SPME has the advantages of being easier, requiring fewer resources, being solventless, and using mild temperatures and small amounts of sample. This limits compound degradation, while being environmentally friendly with less energy requirements and generating less waste. This sample preparation method is established in the literature, the fibres are commercially available, and automation is possible, which can facilitate and reduce costs in operation. 

The intra-day and inter-day precision were evaluated using Cascade hop with HS-SPME-GC×GC–MS selected for 22 compounds and tabulated in Table S2. The intra-day precision (*n* = 5 injections on the same day) displayed RSD % of retention time of 0.00–0.23% for ^1^*t*_R_, 0.05–4.30% for ^2^*t*_R_, and between 1.51% and 9.63% for peak areas. The inter-day precision was analysed with *n* = 9, where three replicates were injected daily over 3 different days with results of 0.22–0.58% for ^1^*t*_R_, 1.21–8.77% for ^2^*t*_R_, and 1.65–12.81% for the area. These results indicate an acceptable repeatability for the proposed method, as compared with the values of Yan et al. [34], which means a low value of RSD even when considering the SPME reproducibility for peak area. 

Compared to previous studies (Table 1), the present methodology resulted in the highest number of peaks tentatively identified in the hop by using GC×GC–MS. The profile of the metabolites present in the five different hops identified numerous peaks by GC×GC–MS, totalling 205 for AZAC, 258 for CASC, 421 for LORA, 472 for ZAPP, and 413 for ENIG. Comparing the number of compounds reported with the GC–MS for the samples (Table 2) proved the improvement in metabolic coverage by GC×GC–MS with increases of 140.2% for CASC, 273.5% for ENIG, and 316.8% for ZAPP. Figure 2 (see Figures S6 and S7 for the other samples) represents aligned peaks with seven metabolites that have significant characteristics for the flavour and odour of hop: (a) β-pinene, (b) β-myrcene, (c) 2-methylbutyl isobutyrate, (d) linalool, (e) geraniol, (f) caryophyllene, and (g) humulene. All these components are now apparently free from interference, although the suite of non-polar compounds could potentially lead to overlapping compounds. Better separation should correspond to improved MS matching with databases. The overall results demonstrate evidence of a better separation, clear distinction of compounds and, consequently, an increase in identified peaks.

The tentative identification was performed using the NIST 11 database, and the highest probability compound identity was determined, considering the MS RMatch (>700) and retention indices (±20 units) based on the van den Dool and Kratz equation (Table S3 and Figures S3–S5). A total of 137 compounds were identified with GC×GC–MS analysis of five hops and a total of 73 compounds for GC–MS analysis of three hops (*n* = 3, Table S4).

The final list of compounds tentatively identified by GC×GC–MS (Table 3) includes their molecular formula, CAS identifier, retention time, retention indices from literature and experiment, the relative chromatographic area of the peaks in each sample, and compound chemical class. Analysis of selected standards was performed for specific compound identification confirmation. The main classes of volatiles identified and the number of compounds were as follows: alcohols (3), aldehydes (4), esters (47), hydrocarbons (7), ketones (9), monoterpene hydrocarbons (17), oxygenated monoterpenes (12), sesquiterpene hydrocarbons (32), and oxygenated sesquiterpenes (3). The total amount of volatiles is expressed in the last line of the table, demonstrating that there remains a small fraction of unknown compounds, from 3.1% to 12.4% of the total area, which could not be identified using the reported criteria. However, this is an improvement in the identified or tentatively identified molecules over previous studies of essential oils since studies such as Wong et al. [33] reported 50.8–90.0% of the total area of the sample by GC×GC.

The most abundant compounds detected using the SPME sampling method were related to the sesquiterpene hydrocarbons humulene (**108**) and caryophyllene (**103**), and the monoterpene hydrocarbon β-myrcene (**22**). These three compounds are reported in the literature as the major components present in hop, being responsible for up to 90% of the composition of hop essential oils [4]. All these substances are formed from precursors obtained through the methyl-D-erythritol 4-phosphate (MEP) pathway followed by the transformation by prenyltransferases into geranyl diphosphate (GPP) [51,52]. β-Myrcene is formed by GPP during hop growth by a monoterpene synthase (MTS2) [52,53] and has the odour characteristics of peppery, spicy, balsam, plastic, and terpene [24]. For the samples studied, the highest area of β-myrcene was reported for the hop ENIG (37.82%), followed by ZAPP (17.06%) and CASC (5.93%). Caryophyllene, or β-caryophyllene, is an isomer of humulene, also identified as α-humulene or α-caryophyllene, and both compounds are produced by sesquiterpene synthase 1 (HlSTS1) from the precursor β-farnesene formed after GPP [52,53]. Humulene is a metabolite that originated in the final stages of hop cone maturation and was reported in the samples with the highest area of 54.07% for the sample LORA, 41.36% for AZAC, and 36.44% for CASC; lowest humulene abundances, although still with a considerable percentage, were 16.04% for ENIG and 10.05% for ZAPP. Caryophyllene showed lower concentrations than humulene in the samples, except for ZAPP, which had twice the caryophyllene area than humulene. Table 3 lists the major compounds in the five hop samples. Another observation noted here and confirmed from the literature was the relation between β-myrcene and humulene, where β-myrcene has an inverse trend in concentration compared to humulene, described by a common intermediate in their biosynthesis via α-acids and β-acids [4,54].

Some compounds have been identified in only one of the samples (i.e., unique to a single hop), with possible use as chemical markers including those in Table 4. 

**Figure 3:**
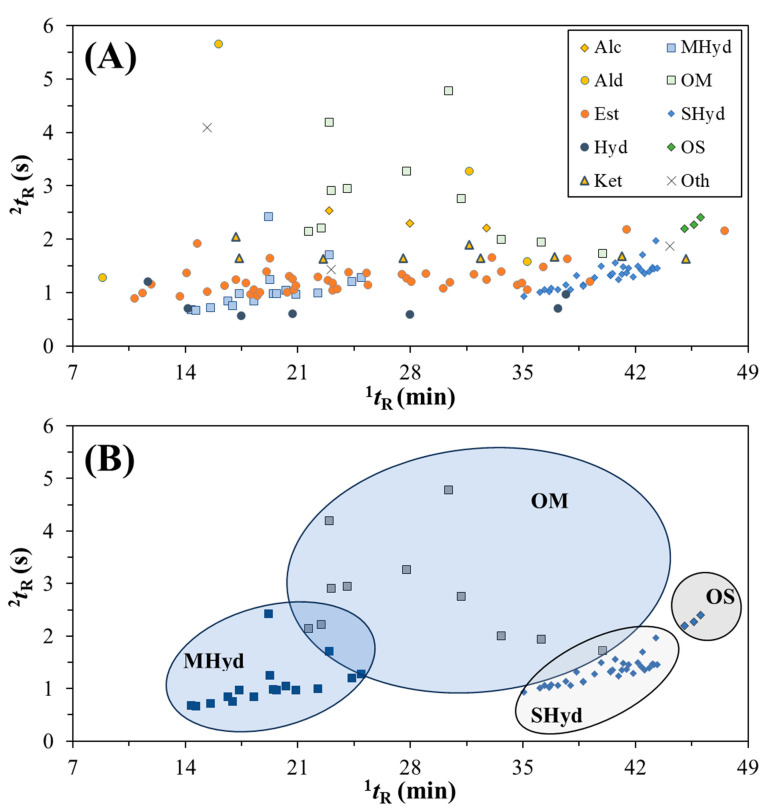
(**A**) Apex plot of the compounds present in the hop samples AZAC, CASC, ENIG, LORA, and ZAPP showing the classes of compounds (refer to Table 3 for abbreviations). (**B**) Apex plot showing clustering of MHyd, OM, SHyd, and OS classes.

The elucidation of the GC×GC distribution of compounds tentatively identified can be expressed by the apex plot in Figure 3a, which was constructed considering all the identified molecules present in Table 3. The classes can be seen as different markers, where the location of peaks shows a region for various classes. For instance, esters (orange circles) persist over the full range of ^1^*t*_R_ but cluster between ^2^*t*_R_ = 1.0 and 2.0 s, and are shown above the hydrocarbons (dark blue circle). The terpenoid compounds, Figure 3b, demonstrate the power of separation of GC×GC through a clear distinction between the classes of MHyd, OM, SHyd, and OS.

**Figure 4:**
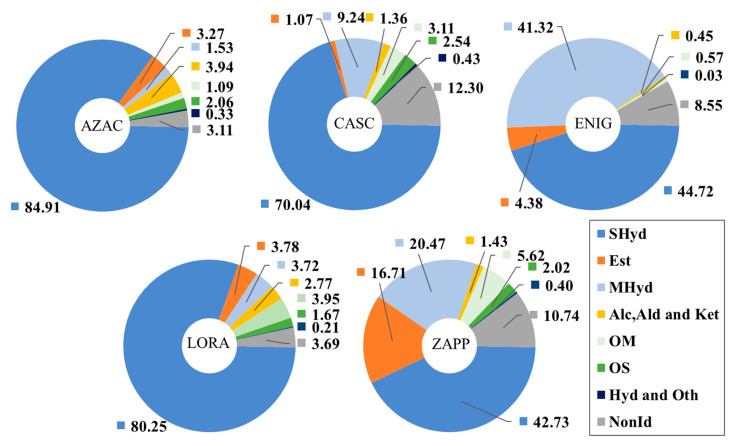
Compositional plots for the hop samples AZAC, CASC, ENIG, LORA, and ZAPP based on the classes of compounds expressed in area percentage (%), according to the SPME methodology employed.

The classes of compounds represented in this study may be considered descriptors of the composition of the hop, displaying the metabolite formation and the identity of the cone. Figure 4 represents the compositional profile of each hop according to the percentage obtained by our methodology (subject to the limitations of reporting peak areas by using SPME), by a clear distinction of the samples. According to Figure 4, all the hops expressed the highest concentration of sesquiterpene hydrocarbons as the major class present, as expected based on the presence of caryophyllene (**103**) and humulene (**108**), with the largest amounts for AZAC, LORA, and CASC, respectively. The hop ENIG and ZAPP expressed a considerable concentration of monoterpene hydrocarbons, which differentiates these from the other hop; this characteristic can be related to the high concentration of β-myrcene (**22**). Furthermore, β-myrcene has a direct relationship with the amount of essential oil produced by the hop with regards to its biosynthetic pathway, giving a different complexity for the hop [4].

**Figure 5:**
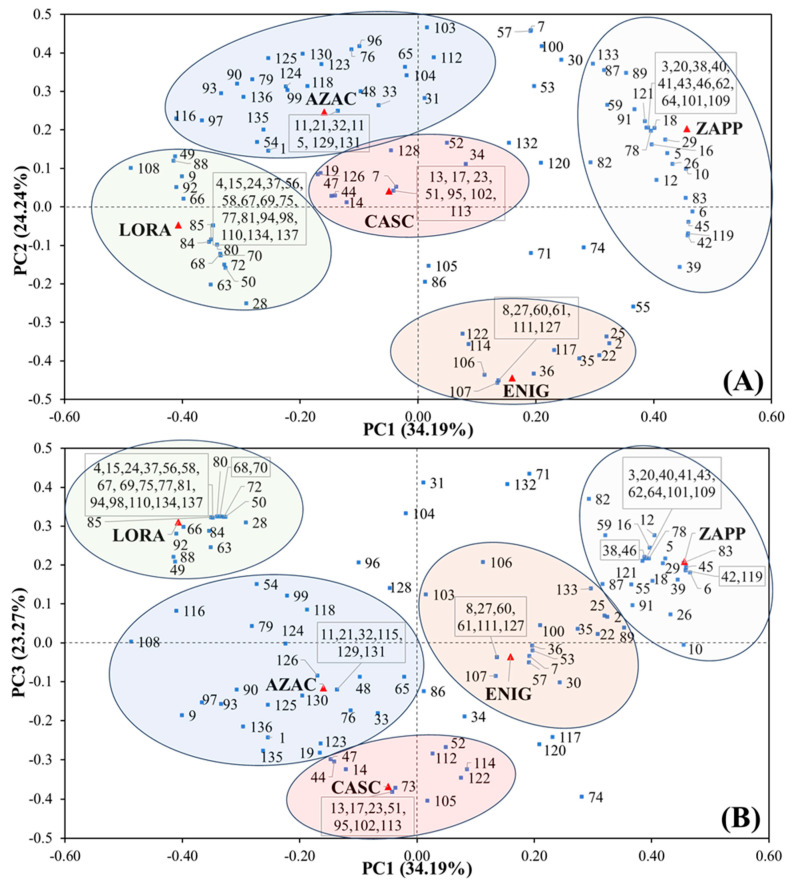
Biplot graph for the principal component analysis (PCA), representing (**A**) PC1×PC2 and (**B**) PC1×PC3. The circles represent the hop samples as AZAC (blue), CASC (pink), ENIG (yellow), LORA (green), and ZAPP (grey).

The amount of data provided by GC×GC illustrates the ease in profiling and describing the sample composition, which should be translated into multivariate statistics, a powerful tool to aid this data interpretation. Considering the data in Table 3, a principal component analysis (PCA) interpretation was applied to the compounds present. For the PCA, the model was built with the relative peak area (%) related to each compound with the selection of autoscale as preprocessing and 3 PCs representing 81.70% of the variance with 34.19% for PC1, 24.24% for PC2, and 23.27% for PC3. The biplot graphs, shown in Figure 5, represent (A) PC1×PC2 and (B) PC1×PC3, where the samples (scores) are represented by red triangles and the compounds (loadings) by blue squares. To improve the view of the compounds related to each sample, coloured regions were drawn for AZAC (blue), CASC (pink), ENIG (yellow), LORA (green), and ZAPP (grey). Regarding this result, a diversity of substances is related to the samples and, as expected, the complexity of compounds formed during hop metabolism. The distribution of compounds can be seen with the ZAPP hop related to the positive part of PC1, while ENIG is present in the negative part of PC2; moreover, AZAC is present in the positive part of PC1 and the negative part of PC2, while PC3 shows LORA in the positive section and CASC in the negative. 

Characterising some of the compounds, attention may be called to the unique compounds in each of the samples with the presence of clusters close to their respective red triangles shown in Figure 5. Characterising each hop variety, AZAC shows the influence of compounds hexanal (**1**), p-cymene (**33**), the sesquiterpene hydrocarbons δ-cadinene (**124**), calamenene (**125**) and α-calacorene (**130**), and the ketones 2-nonanone (**48**), 2-decanone (**65**), 2-undecanone (**79**), 2-dodecanone (**96**) and 2-tridecanone (**116**). This shows a significant connection between AZAC and the ketone formed in its metabolism. Regarding reports from the literature [55], the hop AZAC was studied by GC–MS and a method for the fraction of hydrodistilled essential oil followed by HS-SPME. This study also showed a high concentration of caryophyllene and a high percentage of humulene as reported by results in Table 3. 

For the hop CASC, the monoterpene hydrocarbons camphene (**14**) and perillene (**52**) and the oxygenated monoterpenes geraniol (**73**), *cis*-linalool oxide (**44**), and *trans*-linalool oxide (**47**) were related by PCA analysis. This shows that CASC has a relation with the auto-oxidation of myrcene [56,57] by the formation of geraniol, an important floral odorant in the hop essential oil, as the presence of camphene and linalool oxide in the two isomeric forms, related to the “European hop aroma” [17,57]. The hop CASC is widely studied in the literature, as in the GC×GC-TOFMS study from Yan et al. [34], and by GC–MS from other studies [55]. In the results of Yan et al., the essential oil fraction of CASC was hydrodistilled and as a result also displayed the presence of geraniol and perillene, proving certain similarities. When compared to the composition of humulene (36.44%) and β-myrcene (5.93%) (noting limitations due to the SPME sampling), this paper shows a contrary behaviour compared to the literature [34,55,58], where the concentration of β-myrcene is higher than humulene, which is explained as a distinct sample with a probable cause of low content of β-myrcene and high content of humulene related to the ripening period, more specifically an early harvest or a not-well-ripe hop [4,54].

Regarding ENIG, three classes were significant: monoterpene hydrocarbons comprising β-myrcene (**22**), β-ocimene (**35**), and *trans*-β-ocimene (**36**), the two sesquiterpene hydrocarbons (*E*)-β-farnesene (**107**) and β-eudesmene (**117**), and the esters isobutyl 2-methylbutanoate (**25**) and 2-methylbutyl octanoate (**106**). Low concentrations of β-farnesene has been reported in low concentrations in hop [4,54], and this can be one of the differentiating components of ENIG hop, while the presence of the monoterpene β-myrcene showed the highest concentration, followed by the two forms of β-ocimene. 

LORA composition noted the esters isoamyl isobutanoate (**28**), ethyl heptanoate (**49**), hexyl isobutyrate (**63**), ethyl octanoate (**66**), *trans*-geranic acid methyl ester (**84**), isobutyric acid 1-methyl-octyl ester (**88**), and ethyl *cis*-4-decenoate (**92**). As reported, the major group responsible for the PCA separation was the esters, even in the specific compounds expressed only by the hop LORA. Nevertheless, compounds such as fenchol (**58**), α-terpineol (**67**), perillaldehyde (**77**), and α-citral (**75**) were important monoterpene alcohols and aldehydes for the chemical composition of this hop. 

Finally, ZAPP showed two compounds within the cluster of specific compounds: prenyl isobutyrate (**38**) and methyl 6-methyl heptanoate (**46**); relations with the esters 2-methylbutyl acetate (**5**), isobutyl isobutyrate (**6**), isobutyl butyrate (**12**), 2-methylbutyl propionate (**16**), 2-methylbutyl isobutyrate (**29**), methyl 8-methylnonanoate (**78**), methyl decanoate (**83**), and methyl 3,6-dodecadienoate (**119**); and the monoterpene hydrocarbons γ-terpinene (**42**), isoterpinolene (**45**), and α-muurolene (**118**). ZAPP was similar to the LORA sample, where the ester class had the capacity to represent the separation of samples by PCA.

**Figure 6:**
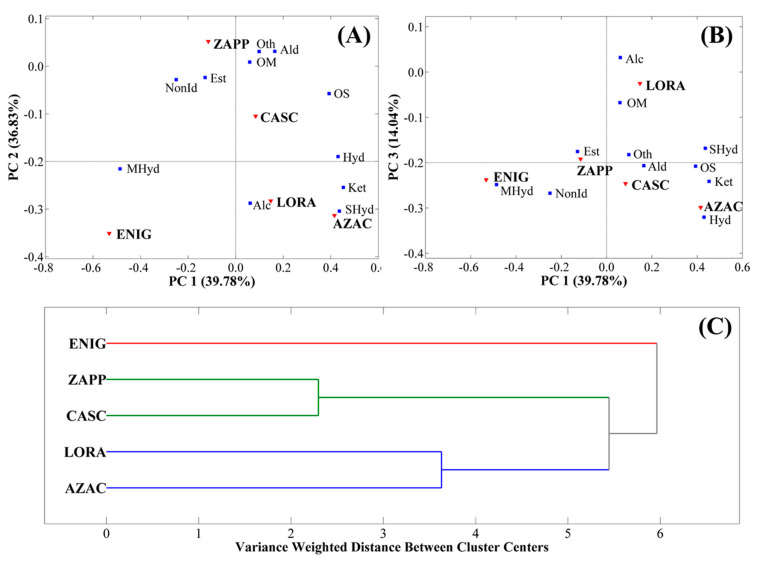
Multivariate statistical analysis of PCA expressed by the biplot graph of (**A**) PC1×PC2 and (**B**) PC1×PC3, and (**C**) HCA for the hops AZAC, CASC, ENIG, LORA, and ZAPP regarding the classes of compounds.

Based on all the information described above and noting some patterns in the PCA (Figure 5), the classes of compounds were also studied according to PCA and HCA. For the PCA, the model was developed with the sum of the relative chromatographic areas (%) of each class using the selection of autoscale as preprocessing and 3 PCs representing 90.58% of variance displaying the variance of 39.78% for PC1, 36.72% for PC2, and 14.08% for PC3. Regarding HCA, autoscale was used as preprocessing, Ward’s method was applied as an algorithm, and PCA was used to choose three PCs. The PCA graphs are expressed as biplot graphs, as shown in Figure 6A (PC1×PC2) and Figure 6B (PC1×PC3), while HCA is represented in Figure 6C. 

The PCA for the classes of compounds separates the ZAPP sample by PC2, LORA by PC3, and ENIG by PC1. As explained previously, this represents that the sample ZAPP was most influenced by esters, LORA by alcohols, and ENIG by monoterpene hydrocarbons, with the influence of ketones over the AZAC hop. HCA gave the highest similarity between classes of ZAPP and CASC, followed by LORA and AZAC, which means that compared to the results shown by PCA, the first two classes were largely influenced by esters, aldehydes, oxygenated monoterpenes, and sesquiterpenes, while the second two were more affected by sesquiterpene hydrocarbons and ketones.

## 4. Conclusions

In this paper, a method for HS-SPME-GC×GC–MS using hop samples was developed, applied, and demonstrated to be a powerful technique to identify metabolites in the samples. By suitable experimental design of the method, we believe this to be a “hop-timal” analysis strategy for total VOC composition in the headspace of the hop cone. The identification of compounds as an untargeted study through this methodology provides considerable coverage of volatile metabolite expression, and it is possible to describe both major and minor compounds, including those most likely not readily measured by single-dimension GC separations since substantially fewer compounds are reported in GC–MS analysis. Thus, the identification of 137 substances in five diverse hop samples is described, with the potential of separating 471 peaks. This of course also highlights that in terms of metabolite identification, just having the ability to separate individual compounds is not the same as being able to unambiguously identify them. In this regard, in comparison with the conventional technique of GC–MS, GC×GC–MS improves the separation through an increase of over 300% in the number of peaks recognised as discrete components. This study reports the comprehensive study of hop through detailed chemical class assignment compounds, which can largely be separated in the 2D space of GC×GC analysis. Multivariate statistics analysis proved a similarity between the samples ZAPP and CASC, and the samples AZAC and LORA. The use of HS-SPME-GC×GC–MS is a bright light in understanding the metabolites present in hop.

## Figures and Tables

**Table 1:** Review table for literature studies of hop by GC×GC.

Hop Types	Sample Preparation	GC×GC Setup	Column Set	Aim of the Study	Compounds	Ref.
Dry-hopped German Pilsner beer using hop pellets of US Eureka harvested in 2016	Degassed beer followed by solvent extraction with diethyl ether, the organic phase dried and applied onto mercurated agarose, and the thiol fraction purified by SAFE	GC×GC–TOFMS, liquid nitrogen-cooled dual-stage quad-jet thermal modulator	^1^D column: DB-FFAP (30 m × 0.25 mm I.D. × 0.25 µm *d*_f_) ^2^D column: DB-5 (2.0 m × 0.15 mm I.D. × 0.30 µm *d*_f_)	Evaluation of the effect of Eureka hop in beers within the process of dry hopping	4MMP	Schmidt et al., 2019 [39]
Dry-hopped Pilsner style beer using hop pellets of US Eureka harvested in 2016 in 4 different days	Degassed beer followed by solvent extraction with diethyl ether, the organic phase dried and applied onto mercurated agarose, and the thiol fraction purified by SAFE	GC×GC–TOFMS, liquid nitrogen-cooled dual-stage quad-jet thermal modulator	^1^D column: DB-FFAP (30 m × 0.25 mm I.D. × 0.25 µm *d*_f_) ^2^D column: DB-5 (2.0 m × 0.15 mm I.D. × 0.30 µm *d*_f_)	Investigation of 4MMP originated from hops during the process of dry hopping and its behaviour through storage	4MMP	Reglitz et al., 2018 [37]
Hop cones from Australia	Hydro-distillation of the cones into essential oil and injection of the diluted solution	GC×GC–QMS, LMCS modulator and GC–GC×GC–accTOFMS, LMCS modulator	QMS ^1^D column: SUPELCOWAX10 (30 m × 0.25 mm I.D. × 0.25 µm *d*_f_) ^2^D column: SLB-IL59 or BPX5 (1.4 m × 0.1 mm I.D. × 0.08 µm *d*_f_) accTOFMS ^1^D column: DB-5 ms (30 m × 0.25 mm I.D. × 0.25 µm *d*_f_) ^2^D column: SUPELCOWAX10 (30 m × 0.25 mm I.D. × 0.25 µm *d*_f_) ^3^D column: DB-5 (1.4 m × 0.1 mm I.D. × 0.08 µm *d*_f_)	Use of a sequential hybrid three-dimensional gas chromatography applied to hop samples	Oxygenated sesquiterpenes in hop and improvement in the separation of those compounds	Yan et al., 2018 [40]
Four experimental hops from Tasmania and 4 commercial hops: Cascade, Galaxy, Helga, and Superpride	Extraction of the essential oils by hydro-distillation with the use of 50 g of dried hops into a Clevenger apparatus	GC×GC–QTOFMS, LMCS modulator	^1^D column: Mega-Wax MS (60 m × 0.25 mm I.D. × 0.25 µm *d*_f_) ^2^D column: BPX5 (2 m × 0.1 mm I.D. × 0.1 µm *d*_f_)	Use of GC×GC–QTOFMS for the comprehensive study of the genotypes present in new hops	210–306 unique compounds were detected and 99 identified	Yan et al., 2018 [34]
78 samples of leaf, wild cones, and hop pellets	Samples were immersed in liquid N_2_, ground into powder, nonvolatiles removed by SAFE, and preconcentration of the solution	GC×GC–TOFMS, liquid nitrogen-cooled dual-stage quad-jet thermal modulator	^1^D column: DB-FFAP (30 m × 0.25 mm I.D. × 0.25 µm *d*_f_) ^2^D column: DB-5 (2.0 m × 0.15 mm I.D. × 0.30 µm *d*_f_)	Development of an analytical method for the determination of 4MSP in hop and hop products	4MSP	Reglitz and Steinhaus, 2017 [41]
Dry hop cones	Hydro-distillation of the cones into essential oil and injection of the diluted solution	2GC×2GC–FID, dual-stage thermal modulator	First parallel setting ^1^D column: BPX5 (60 m × 0.25 mm I.D. × 0.25 µm *d*_f_) ^2^D column: BP10 (1.2 m × 0.25 mm I.D. × 0.25 µm *d*_f_) Second parallel setting ^1^D column: SolGel-Wax (60 m × 0.25 mm I.D. × 0.25 µm *d*_f_) ^2^D column: SolGel-Wax (1.2 m × 0.25 mm I.D. × 0.25 µm *d*_f_)	A new system of parallel comprehensive two-dimensional gas chromatography with the application of hop	-	Yan et al., 2017 [36]
Hop cultivars from four farms in the Saaz region, collected between 2011 and 2014	SPE (C18 500 mg bonded silica sorbent) of hop solutions	GC×GC–TOFMS, quad-jet dual-stage cryo-modulator using liquid nitrogen	^1^D column: DB-WAX (60 m × 0.25 mm I.D. × 0.25 µm *d*_f_) ^2^D column: DB-5ms (1.6 m × 0.18 mm I.D. × 0.18 µm *d*_f_)	Study of the time of harvest and pruning date on aroma characteristics of hop teas	63 compounds were identified and 33 compounds quantified	Inui et al., 2016 [42]
Hallertauer Mittelfrüh, Saazer, Tradition, Perle, and Cascade	Extraction by the mixture of 350 mL of beer and 300 g of CH_2_Cl_2_, separation of the organic phase, and preconcentration	GC×GC–TOFMS, quad-jet dual-stage cryo-modulator using liquid nitrogen	^1^D column: Rtx-1 (30 m × 0.25 mm I.D. × 0.25 µm *d*_f_) ^2^D column: InertCap 17 (1.6 m × 0.10 mm I.D. × 0.1 µm *d*_f_)	Determination of the relationship between key hop-derived compounds and sensorial properties	67 compounds identified	Inui et al., 2013 [24]
American-style lager beer with the addition of light-stable hops	2 cm, 85 µm Car/PDMS SPME fibre for the GC×GC–MS–olfactometry and a 10 mm × 0.5 mm PDMS stir bar (SBSE) for TOFMS	GC×GC–MS–olfactometry, GC×GC–TOFMS, dual-jet thermo modulator	^1^D column: DB-5 (10 m × 0.18 mm I.D. × 0.18 µm *d*_f_) ^2^D column: Rxi-17ms (1.0 m × 0.15 mm I.D. × 0.30 µm *d*_f_)	Study of the flavour changes in beer by the process of oxidation by GC×GC and olfactometry	7 key olfactory compounds	Lusk et al., 2012 [43]
Hop essential oil from the types Target and Cascade	Extraction of hop pellets using liquid CO_2_ and isolation of the essential oil by distillation performed with high vacuum	GC×GC–FID, GC×GC–TOFMS, LMCS modulator	FID ^1^D column: BPX5 (30 m × 0.25 mm I.D. × 0.25 µm *d*_f_) ^2^D column: BP20 (1.1 m × 0.1 mm I.D. × 0.1 µm *d*_f_) TOFMS ^1^D column: BPX5 (30 m × 0.25 mm I.D. × 0.25 µm *d*_f_) ^2^D column: BP20 (0.8 m × 0.1 mm I.D. × 0.1 µm *d*_f_)	Development of a methodology for the identification of compounds with odorant impact	Monoterpene and sesquiterpene alcohols from the spicy fraction and 8 peaks were resolved in a heart-cut of 18s	Eyres, Marriott, and Dufour, 2007 [44]
Hops Target, Saaz, Hallertauer, Hersbrucker, and Cascade	Extraction of hop pellets using liquid CO_2_ and isolation of the essential oil by distillation performed with high vacuum	GC×GC–FID and GC×GC–TOFMS, LMCS modulator	FID ^1^D column: BPX5 (30 m × 0.25 mm I.D. × 0.25 µm *d*_f_) ^2^D column: BP20 (1.1 m × 0.1 mm I.D. × 0.1 µm *d*_f_) TOFMS ^1^D column: BPX5 (30 m × 0.25 mm I.D. × 0.25 µm *d*_f_) ^2^D column: BP20 (0.8 m × 0.1 mm I.D. × 0.1 µm *d*_f_)	Identification of odorants present in the spice fraction of hop essential oils by GC×GC–FID, GC×GC–TOFMS, GC–O, and heart-cut MDGC–O	119 odour-active regions and some compounds identified (14-hydroxy-β-caryophyllene, geraniol, linalool, β-ionone, and eugenol) in one region	Eyres, Marriott, and Dufour, 2007 [38]
Hop essential oil from Target hops	Molecular-distilled, liquid CO_2_ extraction of oil fraction	GC×GC–TOFMS, thermal modulator with cryogenic trapping	^1^D column: DB-5 (10 m × 0.18 mm I.D. × 0.18 µm *d*_f_) ^2^D column: DB17 (1.9 m × 0.1 mm I.D. × 0.1 µm *d*_f_)	Use of GC×GC–TOFMS to separate and identify compounds in hop essential oils	More than 1000 peaks and 119 compounds identified	Roberts, Dufour, and Lewis, 2004 [45]
Hop essential oil	-	GC×GC–FID, LMCS modulator	^1^D column: BPX5 (30 m × 0.25 mm I.D. × 0.25 µm *d*_f_) ^2^D column: BP20 (0.8 m × 0.1 mm I.D. × 0.1 µm *d*_f_)	Determination of compounds in hop essential oil by GC×GC	-	Dufour et al., 2003 [35]

MS—mass spectrometry; QMS—quadrupole MS; LMCS—longitudinally modulated cryogenic system; accTOFMS—accurate mass time-of-flight MS; ^1^D—first dimension; ^2^D—second dimension; QTOFMS—quadrupole time-of-flight MS; 2GC×2GC—parallel comprehensive two-dimensional gas chromatography; 4MMP—4-mercapto-4-methylpentan-2-one; SAFE—solvent-assisted flavour evaporation; TOFMS—time-of-flight MS; 4MSP—4-methyl-4-sulfanylpentan-2-one; SPE—solid phase extraction; Car—carboxen; PDMS—polydimethylsiloxane; SPME—solid phase microextraction; SBSE—stir bar sorptive extraction; FID—flame ionisation detector; O—olfactometry; and MDGC—multidimensional gas chromatography.

**Table 2:** Comparison of peaks from 3 hop samples by GC–MS and GC×GC–MS.

Samples	No. of Peaks of GC–MS		No. of Peaks of GC×GC–MS	
	Integrated	Identified	Integrated	Identified
CASC	184	45	258	67
ENIG	151	51	413	71
ZAPP	149	51	472	81

**Table 3:** The composition of hop samples determined by using HS–SPME–GC×GC–MS.

N.	^1^*t*_R_ (min)	^2^*t*_R_ (s)	Compound *	Class ^a^	CAS	Formula	Lit. RI ^b^	Exp. RI ^c^	Relative GC×GC–MS TIC Area (%)				
									AZAC	CASC	ENIG	LORA	ZAPP
1	8.9	1.28	Hexanal	Ald	66-25-1	C_6_H_12_O	800 ± 2	800	0.005	0.015	ND	0.009	0.002
2	10.9	0.90	Propyl isobutyrate	Est	644-49-5	C_7_H_14_O_2_	842 ± 6	848	ND	ND	0.005	ND	0.002
3	11.4	0.99	Isobutyl propionate	Est	540-42-1	C_7_H_14_O_2_	866 ± 2	859	ND	ND	ND	ND	0.011
4	11.7	1.21	Ethylbenzene	Hyd	100-41-4	C_8_H_10_	855 ± 10	867	ND	ND	ND	0.004	ND
5	11.9	1.16	2-Methylbutyl acetate	Est	624-41-9	C_7_H_14_O_2_	880 ± 3	871	ND	ND	0.007	ND	0.046
6	13.7	0.93	Isobutyl isobutyrate	Est	97-85-8	C_8_H_16_O_2_	910 ± 4	912	0.025	0.032	0.352	ND	0.695
7	14.1	1.36	Methyl hexanoate	Est	106-70-7	C_7_H_14_O_2_	925 ± 3	920	0.023	0.019	ND	0.008	0.030
8	14.2	0.71	(*E*)-1,3-Nonadiene	Hyd	56700-77-7	C_9_H_16_	924 ± 0	922	ND	ND	0.015	ND	ND
9	14.4	0.67	α-Thujene	MHyd	2867-05-2	C_10_H_16_	929 ± 2	927	0.023	0.044	0.006	0.037	ND
10	14.7	0.67	α-Pinene ^d^	MHyd	80-56-8	C_10_H_16_	937 ± 3	933	0.037	0.147	0.106	0.017	0.267
11	14.8	1.93	Methyl 4-methyl-3-pentenoate	Est	2258-65-3	C_7_H_14_O_2_	NA	935	0.023	ND	ND	ND	ND
12	15.4	1.01	Isobutyl butyrate	Est	539-90-2	C_8_H_16_O_2_	955 ± 6	947	ND	ND	0.004	0.002	0.012
13	15.4	4.09	4,4-Dimethyl-2-buten-4-olide	Oth	20019-64-1	C_6_H_8_O_2_	952 ± 5	947	ND	0.086	ND	ND	ND
14	15.6	0.72	Camphene ^d^	MHyd	79-92-5	C_10_H_16_	952 ± 2	951	ND	0.060	0.003	0.014	ND
15	16.1	5.67	Benzaldehyde	Ald	100-52-7	C_7_H_6_O	962 ± 3	961	ND	ND	ND	0.003	ND
16	16.5	1.14	2-Methylbutyl propionate	Est	2438-20-2	C_8_H_16_O_2_	970 ± 4	969	0.010	ND	0.081	0.051	0.706
17	16.7	0.84	β-Thujene	MHyd	28634-89-1	C_10_H_16_	966 ± 12	973	ND	0.006	ND	ND	ND
18	17.0	0.76	β-Pinene ^d^	MHyd	127-91-4	C_10_H_16_	979 ± 2	980	0.041	0.238	0.085	0.057	1.154
19	17.2	2.05	6-Methyl-5-heptene-2-one	Ket	110-93-0	C_8_H_14_O	986 ± 2	984	0.027	0.206	ND	0.077	0.022
20	17.2	1.25	Methyl isoheptanoate	Est	2177-83-5	C_8_H_16_O_2_	993 ± NA	984	ND	ND	ND	ND	0.035
21	17.4	1.64	2-Octanone	Ket	111-13-7	C_8_H_16_O	990 ± 7	988	0.030	ND	ND	ND	ND
22	17.4	0.98	β-Myrcene ^d^	MHyd	123-35-3	C_10_H_16_	991 ± 2	988	0.907	5.931	37.817	2.632	17.065
23	17.5	0.57	2,2,4,6,6-Pentamethylheptane	Hyd	13475-82-6	C_12_H_26_	991 ± 4	990	ND	0.045	ND	ND	ND
24	17.8	1.18	Ethyl hexanoate ^d^	Est	123-66-0	C_8_H_16_O_2_	1000 ± 2	996	ND	ND	ND	0.003	ND
25	18.1	0.97	Isobutyl 2-methylbutanoate	Est	2445-67-2	C_9_H_18_O_2_	1004 ± 4	1002	0.010	0.003	0.060	0.005	0.031
26	18.3	1.06	Isobutyl isovalerate	Est	589-59-3	C_9_H_18_O_2_	1005 ± 2	1006	ND	0.020	0.012	0.003	0.046
27	18.3	0.85	α-Phellandrene	MHyd	99-83-2	C_10_H_16_	1005 ± 2	1006	ND	ND	0.016	ND	ND
28	18.5	0.94	Isoamyl isobutanoate	Est	2050-01-3	C_9_H_18_O_2_	1015 ± 3	1010	ND	ND	0.222	0.503	ND
29	18.7	1.01	2-Methylbutyl isobutyrate	Est	2445-69-4	C_9_H_18_O_2_	1016 ± 2	1014	2.184	0.599	2.185	0.738	7.530
30	19.1	1.40	Methyl heptanoate	Est	106-73-0	C_8_H_16_O_2_	1023 ± 3	1022	0.127	0.061	0.024	ND	0.122
31	19.2	2.43	o-Cymene	MHyd	527-84-4	C_10_H_14_	1022 ± 2	1023	0.007	ND	ND	0.012	0.014
32	19.2	1.64	Methyl 3-methyl-3-hexenoate	Est	50652-84-1	C_8_H_14_O_2_	NA	1025	0.221	ND	ND	ND	ND
33	19.3	1.25	p-Cymene	MHyd	99-87-6	C_10_H_14_	1025 ± 2	1025	0.103	0.284	0.008	0.146	0.156
34	19.5	0.98	D-Limonene ^d^	MHyd	5989-27-5	C_10_H_16_	1030 ± 2	1029	0.229	1.103	0.304	0.497	0.699
35	19.7	0.98	β-Ocimene	MHyd	13877-91-3	C_10_H_16_	1037 ± 7	1033	ND	ND	0.078	ND	0.026
36	20.3	1.05	*trans*-β-Ocimene	MHyd	3779-61-1	C_10_H_16_	1049 ± 2	1045	ND	ND	2.806	ND	0.394
37	20.4	1.01	Amyl isobutyrate	Est	2445-72-9	C_9_H_18_O_2_	1056 ± 1	1047	ND	ND	ND	0.007	ND
38	20.5	1.31	Prenyl isobutyrate	Est	76649-23-5	C_9_H_16_O_2_	1052 ± 1	1049	ND	ND	ND	0.007	0.871
39	20.7	1.25	(*E*)-2-Methylbut-2-en-1-yl isobutyrate	Est	95654-17-4	C_9_H_16_O_2_	1059 ± NA	1053	ND	ND	0.041	ND	0.048
40	20.7	0.61	9-Methyl-1-decene	Hyd	61142-78-7	C_11_H_22_	1055 ± NA	1053	ND	ND	ND	ND	0.011
41	20.8	1.06	2-Methylbutyl butanoate	Est	51115-64-1	C_9_H_18_O_2_	1056 ± 3	1055	ND	ND	ND	ND	0.011
42	20.9	0.97	γ-Terpinene	MHyd	99-85-4	C_10_H_16_	1060 ± 3	1057	ND	ND	0.015	ND	0.023
43	20.9	1.14	Methyl 2-methylheptanoate	Est	51209-78-0	C_9_H_18_O_2_	1067 ± NA	1057	ND	ND	ND	ND	0.010
44	21.7	2.15	*cis*-Linalool oxide	OM	5989-33-3	C_10_H_18_O_2_	1074 ± 4	1073	ND	0.022	ND	0.006	ND
45	22.3	1.00	Isoterpinolene	MHyd	586-63-0	C_10_H_16_	1086 ± 3	1084	ND	ND	0.015	ND	0.028
46	22.3	1.29	Methyl 6-methyl heptanoate	Est	2519-37-1	C_9_H_18_O_2_	NA	1084	ND	ND	ND	0.020	1.968
47	22.5	2.22	*trans*-Linalool oxide	OM	34995-77-2	C_10_H_18_O_2_	1086 ± 5	1088	ND	0.036	ND	0.010	ND
48	22.6	1.63	2-Nonanone	Ket	821-55-6	C_9_H_18_O	1092 ± 2	1090	0.508	0.022	ND	0.028	0.085
49	22.9	1.24	Ethyl heptanoate	Est	106-30-9	C_9_H_18_O_2_	1097 ± 3	1096	0.012	ND	ND	0.016	ND
50	23.0	2.54	2-Nonanol	Alc	628-99-9	C_9_H_20_O	1102 ± 4	1098	ND	ND	0.029	0.125	ND
51	23.0	4.19	Isomyrcenol	OM	6994-89-4	C_10_H_16_O	NA	1098	ND	0.062	ND	ND	ND
52	23.0	1.71	Perillene	MHyd	539-52-6	C_10_H_14_O	1101 ± 2	1098	0.185	1.432	0.035	0.309	0.640
53	23.1	1.43	Hop ether	Oth	344294-72-0	C_10_H_16_O	NA	1100	0.063	0.199	ND	0.089	0.232
54	23.1	2.91	Linalool ^d^	OM	78-70-6	C_10_H_18_O	1099 ± 2	1100	0.635	1.376	0.141	1.952	0.979
55	23.2	1.05	2-Methylbutyl 2-methylbutanoate	Est	2445-78-5	C_10_H_20_O_2_	1105 ± 2	1102	0.077	0.052	0.169	0.068	0.140
56	23.2	1.18	Hexyl propanoate	Est	2445-76-3	C_9_H_18_O_2_	1108 ± 6	1102	ND	ND	ND	0.017	ND
57	23.5	1.07	2-Methylbutyl isovalerate	Est	2445-77-4	C_10_H_20_O_2_	1107 ± 2	1108	0.214	0.197	0.086	0.128	0.255
58	24.1	2.95	Fenchol	OM	1632-73-1	C_10_H_18_O	1113 ± 4	1120	ND	ND	ND	0.003	ND
59	24.2	1.38	Methyl octanoate	Est	111-11-5	C_9_H_18_O_2_	1126 ± 2	1122	0.067	0.034	0.043	0.057	0.142
60	24.4	1.21	Neo-allo-ocimene	MHyd	7216-56-0	C_10_H_16_	1131 ± 0	1126	ND	ND	0.005	ND	ND
61	25.0	1.28	(4*E*,6*E*)-Allocimene	MHyd	3016-19-1	C_10_H_16_	1144 ± 1	1138	ND	ND	0.020	ND	ND
62	25.3	1.38	3-Methylbut-2-en-1-yl pivalate	Est	211429-71-9	C_10_H_18_O_2_	1141 ± NA	1144	ND	ND	ND	ND	0.010
63	25.4	1.14	Hexyl isobutyrate	Est	2349-07-7	C_10_H_20_O_2_	1150 ± 2	1146	0.081	0.001	0.095	0.156	ND
64	27.5	1.34	Methyl 6-methyloctanoate	Est	5129-62-4	C_10_H_20_O_2_	1193 ± 5	1188	ND	ND	ND	ND	0.749
65	27.6	1.64	2-Decanone	Ket	693-54-9	C_10_H_20_O	1193 ± 2	1190	0.489	0.078	ND	0.032	0.182
66	27.8	1.26	Ethyl octanoate	Est	106-32-1	C_10_H_20_O_2_	1196 ± 3	1194	0.007	ND	ND	0.026	ND
67	27.8	3.27	α-Terpineol	OM	98-55-5	C_10_H_18_O	1189 ± 2	1194	ND	ND	ND	0.012	ND
68	28.0	2.29	2-Decanol	Alc	1120-06-5	C_10_H_22_O	1200 ± 7	1198	ND	ND	0.018	0.115	ND
69	28.0	0.60	Dodecane	Hyd	112-40-3	C_12_H_26_	1200 ± NA	1198	ND	ND	ND	0.012	ND
70	28.1	1.20	Heptyl propanoate	Est	2216-81-1	C_10_H_20_O_2_	1201 ± NA	1200	ND	ND	0.003	0.020	ND
71	29.0	1.36	Methyl nonanoate	Est	1731-84-6	C_10_H_20_O_2_	1225 ± 2	1219	ND	ND	0.012	0.015	0.018
72	30.1	1.08	HeptyI isobutyrate	Est	2349-13-5	C_11_H_22_O_2_	1247 ± 1	1243	ND	ND	0.050	0.233	ND
73	30.4	4.78	Geraniol ^d^	OM	106-24-1	C_10_H_18_O	1255 ± 3	1249	0.034	0.893	0.027	0.054	0.066
74	30.5	1.19	2-Methylbutyl hexanoate	Est	2601-13-0	C_11_H_22_O_2_	1247 ± 1	1251	0.012	0.017	0.017	ND	0.011
75	31.2	2.76	α-Citral	OM	141-27-5	C_10_H_16_O	1270 ± 2	1266	ND	ND	ND	0.010	ND
76	31.7	1.89	(*Z*)-Undec-6-en-2-one	Ket	107853-70-3	C_11_H_20_O	1274 ± NA	1277	0.271	0.148	0.028	0.089	0.122
77	31.7	3.28	Perillaldehyde	Ald	2111-75-3	C_10_H_14_O	1272 ± 4	1277	ND	ND	ND	0.011	ND
78	32.0	1.34	Methyl 8-methylnonanoate	Est	5129-54-4	C_11_H_22_O_2_	1277 ± NA	1283	ND	ND	0.007	ND	0.350
79	32.4	1.64	2-Undecanone	Ket	112-12-9	C_11_H_22_O	1294 ± 2	1291	1.935	0.669	0.293	1.244	0.731
80	32.8	2.21	2-Undecanol	Alc	1653-30-1	C_11_H_24_O	1307 ± 4	1300	ND	ND	0.049	0.460	ND
81	32.8	1.24	n-Octyl propionate	Est	142-60-9	C_11_H_22_O_2_	1302 ± NA	1300	ND	ND	ND	0.048	ND
82	33.1	1.65	Methyl (*Z*)-4-decenoate	Est	7367-83-1	C_11_H_20_O_2_	NA	1307	0.108	0.037	0.471	0.956	2.256
83	33.7	1.40	Methyl decanoate	Est	110-42-9	C_11_H_22_O_2_	1325 ± 1	1320	ND	ND	0.014	ND	0.034
84	33.7	2.00	*trans*-Geranic acid methyl ester	OM	1189-09-9	C_11_H_18_O_2_	1324 ± 2	1320	0.251	0.438	0.403	1.785	0.262
85	34.7	1.14	n-Octyl isobutyrate	Est	109-15-9	C_12_H_24_O_2_	1346 ± 3	1342	0.022	ND	0.052	0.537	ND
86	35.0	1.18	Isopentyl heptanoate	Est	109-25-1	C_12_H_24_O_2_	1334 ± 1	1349	0.013	ND	0.015	ND	ND
87	35.1	0.94	α-Cubebene	SHyd	17699-14-8	C_15_H_24_	1351 ± 2	1351	0.024	0.014	ND	0.009	0.060
88	35.3	1.05	Isobutyric acid 1-methyl-octyl ester	Est	69121-76-2	C_13_H_26_O_2_	1365 ± NA	1356	0.012	ND	ND	0.017	ND
89	35.3	1.58	2-Methyl-1-undecanal	Ald	110-41-8	C_12_H_24_O	1365 ± 2	1356	0.024	0.022	ND	ND	0.063
90	36.1	1.01	Ylangene	SHyd	14912-44-8	C_15_H_24_	1372 ± 2	1373	0.680	0.377	0.141	0.398	0.218
91	36.2	1.94	Geranyl acetate ^d^	OM	105-87-3	C_12_H_20_O_2_	1382 ± 3	1376	0.016	0.086	ND	0.016	0.233
92	36.3	1.48	Ethyl *cis*-4-decenoate	Est	7367-84-2	C_12_H_22_O_2_	1361 ± 2	1378	0.025	ND	ND	0.065	ND
93	36.4	1.06	Copaene	SHyd	3856-25-5	C_15_H_24_	1376 ± 2	1380	2.623	1.643	0.567	1.595	0.739
94	36.7	1.02	β-Bourbonene	SHyd	5208-59-3	C_15_H_24_	1384 ± 3	1387	ND	ND	ND	0.027	ND
95	36.8	1.08	α-Bourbonene	SHyd	5208-58-2	C_15_H_24_	1384 ± 8	1389	ND	0.011	ND	ND	ND
96	37.0	1.67	2-Dodecanone	Ket	6175-49-1	C_12_H_24_O	1396 ± 9	1393	0.200	0.056	0.011	0.148	0.159
97	37.2	1.06	(+)-Sativene	SHyd	3650-28-0	C_15_H_24_	1396 ± 0	1398	0.047	0.022	0.005	0.025	ND
98	37.2	0.72	Tetradecane	Hyd	629-59-4	C_14_H_30_	1400 ± NA	1398	ND	ND	ND	0.011	ND
99	37.7	0.97	1,3-Dimethyl-5-n-propyl-adamantane	Hyd	19385-87-6	C_15_H_26_	NA	1409	0.029	ND	ND	0.015	0.008
100	37.7	1.14	Isocaryophyllene	SHyd	118-65-0	C_15_H_24_	1406 ± 3	1410	0.029	0.036	ND	0.021	0.059
101	37.8	1.64	Methyl undecenoate	Est	111-81-9	C_12_H_22_O_2_	1427 ± 2	1412	ND	ND	ND	ND	0.011
102	38.0	1.06	*cis*-α-Bergamotene	SHyd	18252-46-5	C_15_H_24_	1415 ± 3	1417	ND	0.040	ND	ND	ND
103	38.4	1.32	Caryophyllene ^d^	SHyd	87-44-5	C_15_H_24_	1419 ± 3	1426	21.711	13.191	8.484	15.606	20.439
104	38.8	1.14	β-Copaene	SHyd	13744-15-5	C_15_H_24_	1432 ± 3	1436	0.417	ND	0.022	0.368	0.443
105	38.8	1.12	α-Bergamotene	SHyd	17699-05-7	C_15_H_24_	1435 ± 4	1436	ND	2.169	0.929	ND	0.007
106	39.2	1.21	2-Methylbutyl octanoate	Est	67121-39-5	C_13_H_26_O_2_	1449 ± 2	1445	ND	ND	0.020	0.009	0.007
107	39.5	1.28	(*E*)-β-Farnesene	SHyd	28973-97-9	C_15_H_24_	1457 ± 2	1452	ND	1.091	9.024	ND	ND
108	39.9	1.50	Humulene ^d^	SHyd	6753-98-6	C_15_H_24_	1454 ± 3	1462	41.362	36.442	16.036	54.069	10.053
109	40.0	1.73	Geranyl propionate	OM	105-90-8	C_13_H_22_O_2_	1475 ± 3	1464	ND	ND	ND	ND	0.282
110	40.5	1.32	7-epi-α-Cadinene	SHyd	483-75-0	C_15_H_24_	1485 ± 10	1476	ND	ND	ND	2.104	ND
111	40.5	1.33	γ-Selinene	SHyd	515-17-3	C_15_H_24_	1479 ± 6	1476	ND	ND	1.369	ND	ND
112	40.6	1.36	γ-Muurolene	SHyd	30021-74-0	C_15_H_24_	1477 ± 3	1479	4.510	3.129	ND	ND	2.218
113	40.8	1.56	α-Curcumene	SHyd	644-30-4	C_15_H_22_	1483 ± 3	1483	ND	0.090	ND	ND	ND
114	41.0	1.24	(*Z*,*E*)-α-Farnesene	SHyd	26560-14-5	C_15_H_24_	1491 ± 3	1488	ND	0.040	0.045	ND	ND
115	41.2	1.35	Eremophilene	SHyd	10219-75-7	C_15_H_24_	1499 ± 8	1493	0.086	ND	ND	ND	ND
116	41.2	1.68	2-Tridecanone	Ket	593-08-8	C_13_H_26_O	1497 ± 4	1493	0.452	0.146	0.022	0.415	0.066
117	41.3	1.48	β-Eudesmene	SHyd	17066-67-0	C_15_H_24_	1486 ± 3	1495	1.733	3.842	5.830	1.045	2.432
118	41.5	1.37	α-Muurolene	SHyd	31983-22-9	C_15_H_24_	1500 ± NA	1500	2.457	ND	ND	1.042	0.701
119	41.5	2.18	Methyl 3,6-dodecadienoate	Est	16106-01-7	C_13_H_22_O_2_	NA	1500	ND	ND	0.338	ND	0.548
120	41.6	1.45	α-Selinene	SHyd	473-13-2	C_15_H_24_	1494 ± 3	1502	ND	3.506	0.405	ND	2.090
121	41.7	1.56	Geranyl isobutyrate	OM	2345-26-8	C_14_H_24_O_2_	1514 ± 2	1505	0.150	0.133	ND	0.085	3.761
122	41.9	1.29	β-Bisabolene	SHyd	495-61-4	C_15_H_24_	1509 ± 3	1510	ND	0.111	0.109	ND	ND
123	42.2	1.50	γ-Cadinene	SHyd	39029-41-9	C_15_H_24_	1513 ± 2	1517	2.959	2.108	0.481	1.126	1.187
124	42.4	1.42	δ-Cadinene	SHyd	483-76-1	C_15_H_24_	1524 ± 2	1522	3.685	1.261	0.937	1.974	1.463
125	42.5	1.70	Calamenene	SHyd	483-77-2	C_15_H_22_	1523 ± 5	1525	0.863	0.572	0.045	0.458	0.314
126	42.6	1.36	Zonarene	SHyd	41929-05-9	C_15_H_24_	1527 ± NA	1527	0.256	0.059	0.128	0.094	0.055
127	42.9	1.39	Cadine-1,4-diene	SHyd	16728-99-7	C_15_H_24_	1533 ± 4	1535	ND	ND	0.149	ND	ND
128	43.1	1.47	α-Cadinene	SHyd	24406-05-1	C_15_H_24_	1538 ± 1	1540	ND	0.205	ND	0.225	0.193
129	43.2	1.45	(4a*R*,8a*S*)-4a-Methyl-1-methylene-7-(propan-2-ylidene)decahydronaphthalene	SHyd	58893-88-2	C_15_H_24_	1544 ± NA	1542	0.970	ND	ND	ND	ND
130	43.3	1.97	α-Calacorene	SHyd	21391-99-1	C_15_H_20_	1542 ± 3	1545	0.151	0.079	0.011	0.065	0.058
131	43.4	1.45	Selina-3,7(11)-diene	SHyd	6813-21-4	C_15_H_24_	1542 ± 3	1547	0.345	ND	ND	ND	ND
132	44.2	1.87	(Z)-Tetradec-6-en-2-one	Oth	NA	C_14_H_26_O	1570 ± NA	1567	ND	ND	ND	0.031	0.047
133	45.1	2.19	Caryophyllene oxide ^d^	OS	1139-30-6	C_15_H_24_O	1581 ± 2	1590	0.593	0.431	ND	0.291	1.427
134	45.2	1.63	2-Tetradecanone	Ket	2345-27-9	C_14_H_28_O	1597 ± 1	1593	ND	ND	ND	0.017	ND
135	45.7	2.28	Humulene epoxide I	OS	19888-33-6	C_15_H_24_O	1604 ± 3	1605	0.120	0.245	ND	0.127	0.040
136	46.1	2.40	Humulene epoxide II	OS	19888-34-7	C_15_H_24_O	1606 ± 2	1616	1.347	1.867	ND	1.254	0.549
137	47.6	2.16	(*E*,*Z*)-5,7-Dodecadien-1-ol acetate	Est	78350-11-5	C_14_H_24_O_2_	1653 ± 0	1657	ND	ND	ND	0.068	ND
								Total	96.889	87.617	91.454	96.282	89.225

Abbreviations: ^1^*t*_R_—retention time in the first dimension; ^2^*t*_R_—retention time in the second dimension; RI—retention index; AZAC—Azacca; CASC—Cascade; ENIG—Enigma; LORA—Loral; ZAPP—Zappa; NA—not applicable or not found; ND—not detected. * Tentative identification. ^a^ Classes: *Alc*—alcohol; *Ald*—aldehyde; *Est*—ester; *Hyd*—hydrocarbon; *Ket*—ketone; *MHyd*—monoterpene hydrocarbon; *OM*—oxygenated monoterpene; *OS*—oxygenated sesquiterpene; *SHyd*—sesquiterpene hydrocarbon; and *Oth*—others. ^b^ Lit. RI—literature retention indexes for the compounds on a semi-standard non-polar column, 5%-phenyl using NIST 11 library and NIST website. ^c^ Exp. RI—experimental retention index calculated by the van den Dool and Kratz equation. ^d^ Identification confirmed by standards analysis.

**Table 4:** Suggested unique marker compounds in each of the hop samples.

Sample	Compounds (Peak Number)
AZAC	methyl 4-methyl-3-pentenoate (**11**), 2-octanone (**21**), methyl 3-methyl-3-hexenoate (**32**), eremophilene (**115**), (4a*R*,8a*S*)-4a-methyl-1-methylene-7-(propan-2-ylidene)decahydronaphthalene (**129**) *, and selina-3,7(**11**)-diene (**131**);
CASC	4,4-dimethyl-2-buten-4-olide (**13**), β-thujene (**17**), 2,2,4,6,6-pentamethylheptane (**23**), isomyrcenol (**51**), α-bourbonene (**95**), cis-α-bergamotene (**102**), and α-curcumene (**113**);
ENIG	(*E*)-1,3-nonadiene (**8**), α-phellandrene (**27**), neo-allo-ocimene (**60**), (4*E*,6*E*)-allocimene (**61**), γ-selinene (**111**), and cadine-1,4-diene (**127**);
LORA	amyl isobutyrate (**37**), hexyl propanoate (**56**), fenchol (**58**), α-terpineol (**67**), dodecane (**69**), α-citral (**75**), perillaldehyde (**77**), n-octyl propionate (**81**), β-bourbonene (**94**), tetradecane (**98**), 7-epi-α-cadinene (**110**), 2-tetradecanone (**134**), and (*E*,*Z*)-5,7-dodecadien-1-ol acetate (**137**);
ZAPP	isobutyl propionate (**3**), methyl isoheptanoate (**20**), 9-methyl-1-decene (**40**), 2-methylbutyl butanoate (**41**), methyl 2-methylheptanoate (**43**), 3-methylbut-2-en-1-yl pivalate (**62**), methyl 6-methyloctanoate (**64**), methyl undecenoate (**101**), and geranyl propionate (**109**).

* Even though an enantioselective column was not used, and so no chirality of a compound can be assessed, this was the entry returned by the database search.

## Data Availability

The raw data supporting the conclusions of this article will be made available by the authors on request.

## Supplementary Materials

The following supporting information (abbreviated titles here) can be downloaded at: https://www.mdpi.com/article/10.3390/metabo14040237/s1. Table S1: Hop samples information; Table S2: Intra-day and inter-day precision of selected peaks; Figure S1: A comparison between two chromatographic settings for GC×GC; Table S3: n-Alkanes the series C8-C21 and their respective retention times; Figure S2: Retention time vs n-alkanes series (Cn) plot; Figure S3: GC×GC–MS chromatogram for the HS–SPME of the series of n-alkanes (C8-C15); Figure S4: GC×GC–MS chromatogram for the HS–SPME of the series of n-alkanes (C16-C21); Figure S5: Comparison of chromatograms obtained for the Enigma (ENIG) hop by HS–SPME; Figure S6: Comparison of chromatograms obtained for the Zappa (ZAPP) hop by HS-SPME; Table S4: Information in the hop composition profile using HS-SPME-GC–MS; Figure S7: GC×GC-MS chromatogram for the HS-SPME of Azacca (AZAC) hop; Figure S8: GC×GC-MS chromatogram for the HS-SPME of Loral (LORA) hop.

## References

[1] Moir M. (2018). Hops—A millennium review. J. Am. Soc. Brew. Chem..

[2] Steenackers B., De Cooman L., De Vos D. (2015). Chemical transformations of characteristic hop secondary metabolites in relation to beer properties and the brewing process: A review. Food Chem..

[3] Research M. Global Hops Market—2023–2030. https://www.marketresearch.com/DataM-Intelligence-4Market-Research-LLP-v4207/Global-Hops-34833012/.

[4] Almaguer C., Schönberger C., Gastl M., Arendt E.K., Becker T. (2014). *Humulus lupulus*—A story that begs to be told. A review. J. Inst. Brew..

[5] Cattoor K., Dresel M., De Bock L., Boussery K., Van Bocxlaer J., Remon J.-P., De Keukeleire D., Deforce D., Hofmann T., Heyerick A. (2013). Metabolism of hop-derived bitter acids. J. Agric. Food Chem..

[6] Knez Hrnčič M., Španinger E., Košir I.J., Knez Ž., Bren U. (2019). Hop compounds: Extraction techniques, chemical analyses, antioxidative, antimicrobial, and anticarcinogenic effects. Nutrients.

[7] Rettberg N., Biendl M., Garbe L.-A. (2018). Hop aroma and hoppy beer flavor: Chemical backgrounds and analytical tools—A review. J. Am. Soc. Brew. Chem..

[8] King A.J., Dickinson J.R. (2003). Biotransformation of hop aroma terpenoids by ale and lager yeasts. FEMS Yeast Res..

[9] Guimarães B.P., Nascimento P.G.B.D., Ghesti G.F. (2021). Intellectual property and plant variety protection: Prospective study on hop (*Humulus lupulus* L.) cultivars. World Patent Inf..

[10] Afendi F.M., Okada T., Yamazaki M., Hirai-Morita A., Nakamura Y., Nakamura K., Ikeda S., Takahashi H., Altaf-Ul-Amin M., Darusman L.K. (2012). KNApSAcK family databases: Integrated metabolite-plant species databases for multifaceted plant research. Plant Cell Physiol..

[11] Nezi P., Cicaloni V., Tinti L., Salvini L., Iannone M., Vitalini S., Garzoli S. (2022). Metabolomic and proteomic profile of dried hop Inflorescences (*Humulus lupulus* L. cv. Chinook and cv. Cascade) by SPME-GC-MS and UPLC-MS-MS. Separations.

[12] Fiehn O., Kopka J., Dormann P., Altmann T., Trethewey R.N., Willmitzer L. (2000). Metabolite profiling for plant functional genomics. Nat. Biotech..

[13] Hughey C.A., McMinn C.M., Phung J. (2016). Beeromics: From quality control to identification of differentially expressed compounds in beer. Metabolomics.

[14] Ikhalaynen Y.A., Plyushchenko I.V., Rodin I.A. (2022). Hopomics: *Humulus lupulus* brewing cultivars classification based on LC-MS profiling and nested feature selection. Metabolites.

[15] Brendel R., Schwolow S., Rohn S., Weller P. (2020). Gas-phase volatilomic approaches for quality control of brewing hops based on simultaneous GC-MS-IMS and machine learning. Anal. Bioanal. Chem..

[16] Fiehn O. (2016). Metabolomics by gas chromatography-mass spectrometry: Combined targeted and untargeted profiling. Curr. Protocols Mol. Biol..

[17] Eyres G., Dufour J.-P., Preedy V.R. (2009). Hop essential oil: Analysis, chemical composition and odor characteristics. Beer in Health and Disease Prevention.

[18] Aberl A., Coelhan M. (2012). Determination of volatile compounds in different hop varieties by headspace-trap GC/MS—In comparison with conventional hop essential oil analysis. J. Agric. Food Chem..

[19] Jorge K., Trugo L.C. (2003). Discrimination of different hop varieties using headspace gas chromatographic data. J. Brazil. Chem. Soc..

[20] Liu Z., Wang L., Liu Y. (2018). Rapid differentiation of Chinese hop varieties (*Humulus lupulus*) using volatile fingerprinting by HS-SPME-GC-MS combined with multivariate statistical analysis. J. Sci. Food Agric..

[21] Eri S., Khoo B.K., Lech J., Hartman T.G. (2000). Direct thermal desorption-gas chromatography and gas chromatography-mass spectrometry profiling of hop (*Humulus lupulus* L.) essential oils in support of varietal characterization. J. Agric. Food Chem..

[22] Kovačevič M., Kač M. (2001). Solid-phase microextraction of hop volatiles. Potential use for determination and verification of hop varieties. J. Chromatogr. A.

[23] Kishimoto T., Teramoto S., Fujita A., Yamada O. (2020). Principal component analysis of hop-derived odorants identified by stir bar sorptive extraction method. J. Am. Soc. Brew. Chem..

[24] Inui T., Tsuchiya F., Ishimaru M., Oka K., Komura H. (2013). Different beers with different hops. Relevant compounds for their aroma characteristics. J. Agric. Food Chem..

[25] Richter T.M., Eyres G.T., Silcock P., Bremer P.J. (2017). Comparison of four extraction methods for analysis of volatile hop-derived aroma compounds in beer. J. Sep. Sci..

[26] Sun S., Wang X., Yuan A., Liu J., Li Z., Xie D., Zhang H., Luo W., Xu H., Liu J. (2022). Chemical constituents and bioactivities of hops (*Humulus lupulus* L.) and their effects on beer-related microorganisms. Food Energ. Sec..

[27] Rubiolo P., Sgorbini B., Liberto E., Cordero C., Bicchi C. (2010). Essential oils and volatiles: Sample preparation and analysis. A review. Flav. Fragr. J..

[28] Abolghasemi M.M., Piryaei M. (2020). Development of direct microwave desorption/gas chromatography mass spectrometry system for rapid analysis of volatile components in medicinal plants. J. Sep. Sci..

[29] Priest M.A., Boersma J.A., Bronczyk S.A. (2018). Effects of aging on hops and liquid CO_2_ hop extracts. J. Am. Soc. Brew. Chem..

[30] Gonçalves J.L., Figueira J.A., Rodrigues F.P., Ornelas L.P., Branco R.N., Silva C.L., Camara J.S. (2014). A powerful methodological approach combining headspace solid phase microextraction, mass spectrometry and multivariate analysis for profiling the volatile metabolomic pattern of beer starting raw materials. Food Chem..

[31] Nolvachai Y., Amaral M.S.S., Herron R., Marriott P.J. (2023). Solid phase microextraction for quantitative analysis—Expectations beyond design?. Green Anal. Chem..

[32] Nolvachai Y., Amaral M.S.S., Marriott P.J. (2023). Foods and contaminants analysis using multidimensional gas chromatography: An update of recent studies, technology, and applications. Anal. Chem..

[33] Wong Y.F., Perlmutter P., Marriott P.J. (2017). Untargeted metabolic profiling of *Eucalyptus* spp. leaf oils using comprehensive two-dimensional gas chromatography with high resolution mass spectrometry: Expanding the metabolic coverage. Metabolomics.

[34] Yan D., Wong Y.F., Tedone L., Shellie R.A., Marriott P.J., Whittock S.P., Koutoulis A. (2018). Chemotyping of new hop (*Humulus lupulus* L.) genotypes using comprehensive two-dimensional gas chromatography with quadrupole accurate mass time-of-flight mass spectrometry. J. Chromatogr. A.

[35] Dufour J.-P., Marriott P.J., Reboul E., Leus M., Silcock P., Quéré J.L., Étiévant P.X. (2002). Quantitative analysis of complex flavour mixtures using comprehensive multidimensional gas chromatography. Proceedings of the Flavour Research at the Dawn of the 21st Century.

[36] Yan D., Tedone L., Koutoulis A., Whittock S.P., Shellie R.A. (2017). Parallel comprehensive two-dimensional gas chromatography. J. Chromatogr. A.

[37] Reglitz K., Lemke N., Steinhaus M., Hanke S. (2018). On the behavior of the important hop odorant 4-mercapto-4-methylpentan-2-one (4MMP) during dry hopping and during Storage of dry hopped beer. Brew. Sci..

[38] Eyres G.T., Marriott P.J., Dufour J.-P. (2007). Comparison of odor-active compounds in the spicy fraction of hop (*Humulus lupulus* L.) essential oil from four different varieties. J. Agric. Food Chem..

[39] Schmidt C., Biendl M., Hanke S., Reglitz K., Steinhaus M. (2019). Dry hopping potential of Eureka! A new hop variety. Brew. Sci..

[40] Yan D., Wong Y.F., Whittock S.P., Koutoulis A., Shellie R.A., Marriott P.J. (2018). Sequential hybrid three-dimensional gas chromatography with accurate mass spectrometry: A novel tool for high-resolution characterization of multicomponent samples. Anal. Chem..

[41] Reglitz K., Steinhaus M. (2017). Quantitation of 4-Methyl-4-sulfanylpentan-2-one (4MSP) in hops by a stable isotope dilution assay in combination with GCxGC–TOFMS: Method development and application to study the influence of variety, Provenance, harvest year, and processing on 4MSP concentrations. J. Agric. Food Chem..

[42] Inui T., Matsui H., Hosoya T., Kumazawa S., Fukui N., Oka K. (2016). Effect of harvest time and pruning date on aroma characteristics of hop teas and related compounds of Saaz hops. J. Am. Soc. Brew. Chem..

[43] Lusk L.T., Kay S.B., Porubcan A., Ryder D.S. (2012). Key olfactory cues for beer oxidation. J. Am. Soc. Brew. Chem..

[44] Eyres G., Marriott P.J., Dufour J.-P. (2007). The combination of gas chromatography-olfactometry and multidimensional gas chromatography for the characterisation of essential oils. J. Chromatogr. A.

[45] Roberts M.T., Dufour J.P., Lewis A.C. (2004). Application of comprehensive multidimensional gas chromatography combined with time-of-flight mass spectrometry (GC x GC-TOFMS) for high resolution analysis of hop essential oil. J. Sep. Sci..

[46] Su X., Yin Y. (2021). Aroma characterization of regional Cascade and Chinook hops (*Humulus lupulus* L.). Food Chem..

[47] Amaral M.S.S., Hearn M.T.W., Marriott P.J. (2023). Lipase-catalysed changes in essential oils revealed by comprehensive two-dimensional gas chromatography. Anal. Bioanal. Chem..

[48] Marriott P.J., Massil T., Hugel H. (2004). Molecular structure retention relationships in comprehensive two-dimensional gas chromatography. J. Sep. Sci..

[49] Marriott P.J., Resende G.A.P. (2023). GC×GC technology for non-targeted analysis of volatile compounds It just makes sense. Wiley Analyt. Sci. Mag..

[50] Wong Y.F., Marriott P.J. (2017). Approaches and challenges for analysis of flavor and fragrance volatiles. J. Agric. Food Chem..

[51] Champagne A., Boutry M. (2017). A comprehensive proteome map of glandular trichomes of hop (*Humulus lupulus* L.) female cones: Identification of biosynthetic pathways of the major terpenoid-related compounds and possible transport proteins. Proteomics.

[52] Eriksen R.L., Padgitt-Cobb L.K., Townsend M.S., Henning J.A. (2021). Gene expression for secondary metabolite biosynthesis in hop (*Humulus lupulus* L.) leaf lupulin glands exposed to heat and low-water stress. Sci. Rep..

[53] Wang G., Tian L., Aziz N., Broun P., Dai X., He J., King A., Zhao P.X., Dixon R.A. (2008). Terpene biosynthesis in glandular trichomes of hop. Plant Physiol..

[54] Howard G.A., Slater C.A. (1957). Evaluation of hops VII. Composition of the essential oil of hops. J. Inst. Brew..

[55] Móricz Á.M., Bartoszek M., Polak J., Marczewska P., Knaś M., Böszörményi A., Fodor J., Kowalska T., Sajewicz M. (2023). A comparison of quantitative composition and bioactivity of oils derived from seven North American varieties of hops (*Humulus lupulus* L.). Separations.

[56] Stevens R. (1967). The chemistry of hop constituents. Chem. Rev..

[57] Rutnik K., Knez Hrnčič M., Jože Košir I. (2021). Hop essential oil: Chemical composition, extraction, analysis, and applications. Food Rev. Int..

[58] Buglass A.J., Caven-Quantrill D.J., Baines D., Seal R. (2012). Applications of natural ingredients in alcoholic drinks. Natural Food Additives, Ingredients and Flavourings.

