# Supplementary material for: Comprehensive Two-Dimensional Gas Chromatography–Mass Spectrometry as a Tool for the Untargeted Study of Hop and Their Metabolites
Source: Metabolites. 2024 Apr 19;14(4):237. doi: 10.3390/metabo14040237 (PMC11051809; doi:10.3390/metabo14040237)

Supplementary Information

Comprehensive two-dimensional gas chromatography as a tool for the study of hops and their metabolites

Glaucimar A.P. Resende[1,2], Michelle S.S. Amaral[1], Bruno G. Botelho[2] and Philip J. Marriott[1]*

[1] Australian Centre for Research on Separation Science, School of Chemistry, Monash University, Clayton, 3800 Australia.

[2] Chemistry Department, Universidade Federal de Minas Gerais, Belo Horizonte, 31270-901, Minas Gerais, Brazil.

*philip.marriott@monash.edu

Metabolites

Table of contents

Table S1. Hop samples information……………………………………………………………..…. 2

Table S2. Intra-day and inter-day precision of selected peaks by HS-SPME-GC×GC–MS in hops……. 3

Figure S1. A comparison between two chromatographic settings for GC×GC applying HS-SPME for the hop Cascade, where the 1D column is a DB-5ms UI (30 m × 0.25 mm I.D. × 0.25 µm df) and the 2D columns: (A) BPX50(1.0 m × 0.10 mm I.D. × 0.10 µm df) and (B) SupelcoWax10 (1.0 m × 0.10 mm I.D. × 0.10 µm df) ……………………………………………………………………………………… 4

Figure S2. Chromatograms of 4 types of SPME fibers, by their color in Cascade hops via HS-SPME-GC×GC–MS ………………………………………………………………………………………. 5

Table S3. n-Alkanes the series C8-C21 and their respective retention times in the first ($^{1}t_R$ – DB-5msUI) and second ($^{2}t_R$ – SUPELCOWAX 10) retention times ……………………………………………… 6

Figure S3. Retention time vs n-alkanes series (Cn) plot for the calculation of the retention index by Van den Dol and Kratz equation ………………………………………………………………………... 6

Figure S4. GC×GC–MS chromatogram for the HS–SPME of the series of n-alkanes (C8-C15) …….... 7

Figure S5. GC×GC–MS chromatogram for the HS–SPME of the series of n-alkanes (C16-C21) …….. 7

Figure S6. Comparison of chromatograms obtained for the Enigma (ENIG) hop by HS–SPME using (A) GC–MS and (B) GC×GC–MS followed by the identification of selected peaks (C) …………………… 8

Figure S7. Comparison of chromatograms obtained for the Zappa (ZAPP) hop by HS-SPME using (A) GC–MS and (B) GC×GC–MS followed by the (C) identification of selected peaks …………………….. 9

Table S4. Information in the hop composition profile using HS-SPME-GC–MS …………….……… 10

Figure S8. GC×GC-MS chromatogram for the HS-SPME of Azacca (AZAC) hop……………………... 13

Figure S9 GC×GC-MS chromatogram for the HS-SPME of Loral (LORA) hop ……………………… 13

Table S1. Hop samples information.

	**Azacca (AZAC)**	**Cascade (CASC)**	**Enigma™ (ENIG)**	**Loral™ (LORA)**	**Zappa (ZAPP)**
Class	Dual Purpose	Aroma	Dual Purpose	Dual Purpose	Aroma
Synonym	Azaaca	-	-	HBC291	-
Purpose	Aroma and Bittering	Aroma	Aroma and Bittering	Aroma and Bittering	Aroma
Heritage	Toyomidori and ADHA 94/95	Cross of english fuggle with a male descendant of the Russia variety Serebrianka	Descendant of Swiss Tettnang hop and North America Hop	Daughter of US Glacier & US Nugget	Unknown
Pellet physical description	T-90 hop pellets (90% weight of the hop cone is maintained), size of the pellet is variable, it has a cylindrical shape and diameter of approximately 6 mm				
Consistency	A solid pellet which has an easy detection of aroma, and it can be break in powder				
*Pelletising temperature**	<55 °C				
*Solubility in water**	Insoluble	Insoluble	Insoluble	Insoluble	Insoluble
*Melting temperature (°C)**	No data available	No data available	No data available	No data available	No data available
*Total oil (mL/100g) **	1.6 - 2.5	0.8 – 2.5	0.8 – 3.2	1.5 – 3.4	1.8 – 2.5

*Information from the supplier

Table S2. Intra-day and inter-day precision of selected peaks by HS-SPME-GC×GC–MS in hops.

No.	Compound	Intra-day R.S.D (%) (n=5)			Inter-day R.S.D (%) (n=9)		
		${}^{1}t_R$	${}^{2}t_R$	Area%	${}^{1}t_R$	${}^{2}t_R$	Area%
1	Isobutyl isobutyrate	0.00	0.05	9.63	0.54	1.21	12.81
2	β-Myrcene	0.00	0.16	3.00	0.58	3.17	10.39
3	Isobutyl 2-methylbutanoate	0.00	0.49	6.73	0.54	4.39	14.13
4	D-Limonene	0.23	4.30	3.12	0.50	5.45	4.43
5	Perillen	0.00	0.47	5.86	0.44	8.67	8.02
6	Linalool	0.00	0.30	5.89	0.44	7.71	6.58
7	2-Undecanone	0.00	0.28	1.56	0.31	5.48	5.61
8	trans-Geranic acid methyl ester	0.00	0.24	1.85	0.30	8.02	7.11
9	Copaene	0.00	0.27	3.69	0.28	3.89	3.87
10	Caryophyllene	0.00	0.69	1.55	0.26	5.97	2.27
11	α-Bergamotene	0.00	0.39	1.87	0.26	3.54	1.65
12	(E)-β-Famesene	0.00	0.27	4.88	0.25	5.48	4.16
13	γ-Muurolene	0.00	0.60	1.78	0.25	5.63	2.24
14	α-Curcumene	0.00	0.25	2.66	0.25	6.68	3.06
15	β-Eudesmene	0.00	0.26	5.27	0.24	7.00	6.32
16	α-Selinene	0.00	0.18	3.18	0.24	5.97	4.58
17	Geranyl isobutyrate	0.00	0.33	1.51	0.22	4.41	2.79
18	β-Bisabolene	0.00	0.22	4.57	0.24	4.80	4.19
19	Calamenene	0.00	0.26	2.53	0.24	6.68	2.76
20	Zonarene	0.00	0.24	8.56	0.24	5.08	8.04
21	α-Cadinene	0.00	0.45	2.13	0.23	6.14	1.87
22	Humulene epoxide I	0.00	0.29	7.85	0.22	8.77	10.15

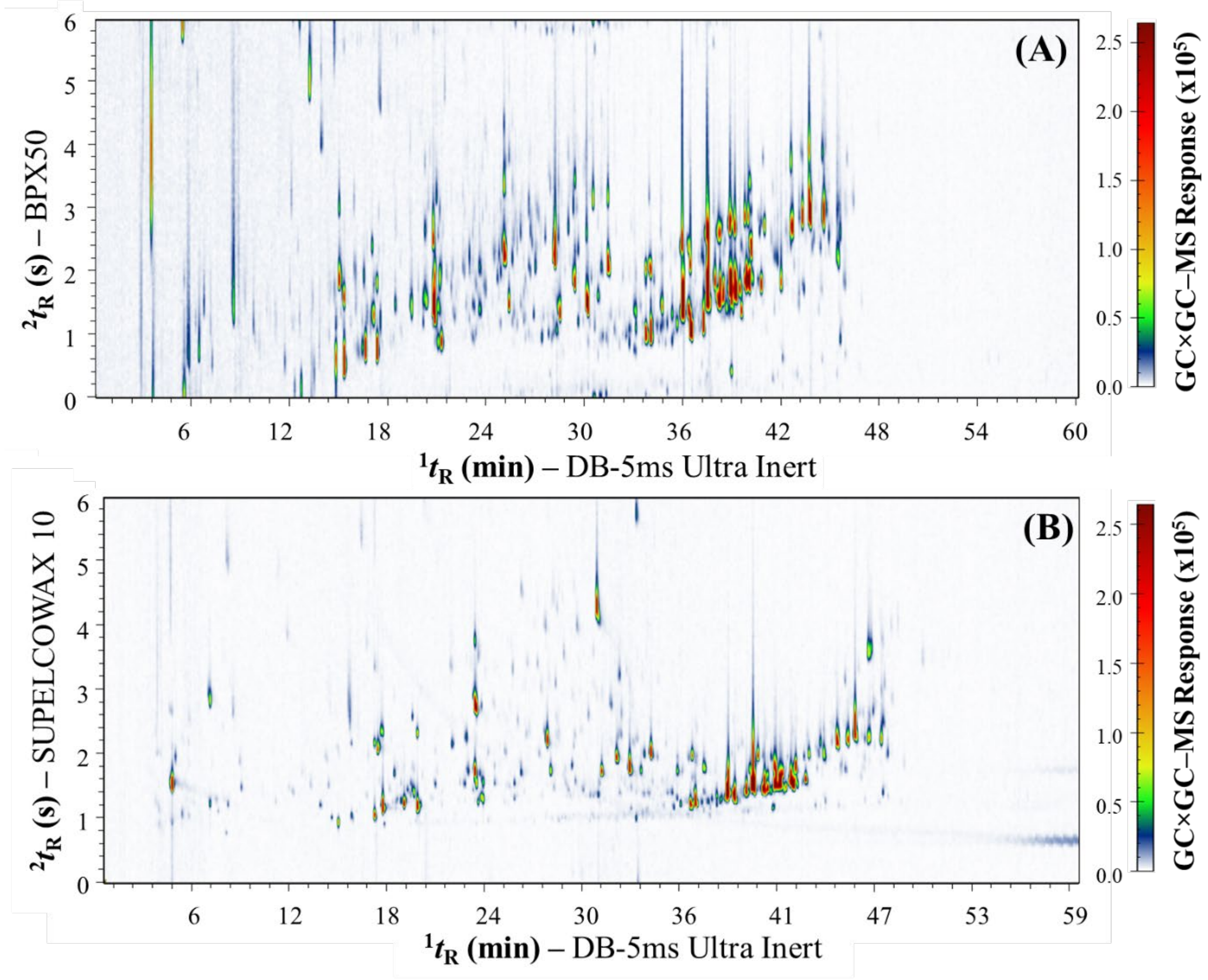

Figure S1. A comparison between two chromatographic settings for GC×GC applying HS-SPME for the hop Cascade, where the 1D column is a DB-5ms UI (30 m × 0.25 mm I.D. × 0.25 µm df) and the 2D columns: (A) BPX50(1.0 m × 0.10 mm I.D. × 0.10 µm df) and (B) SupelcoWax10 (1.0 m × 0.10 mm I.D. × 0.10 µm df)

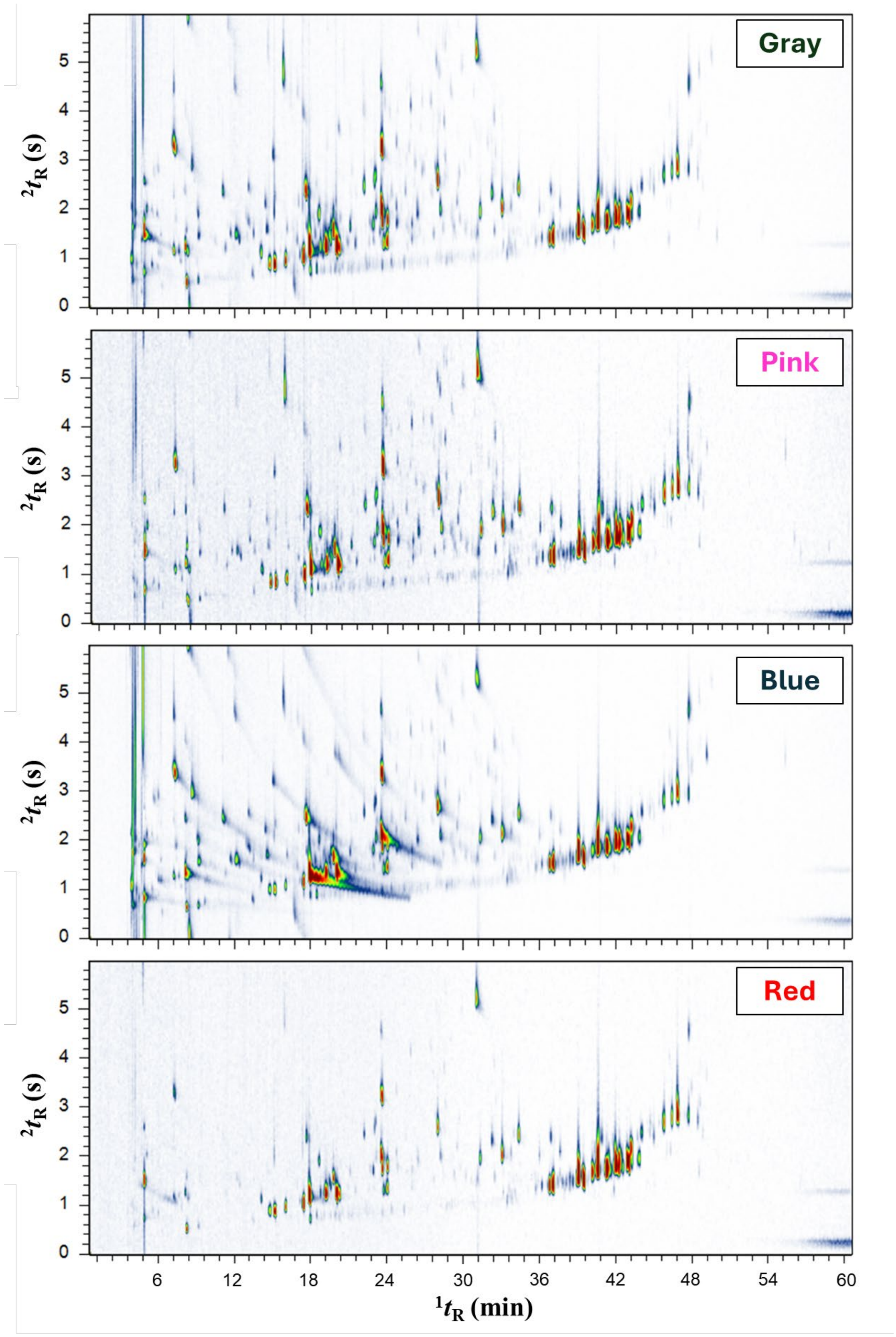

Figure S2. Chromatograms of 4 types of SPME fibres, by their color in Cascade hops by HS-SPME-GC×GC–MS.

Table S3. n-Alkanes the series C8-C21 and their respective retention times in the first ($^{1}t_R$ – DB-5msUI) and second ($^{2}t_R$ – SUPELCOWAX 10) retention times

Alkane	$^{1}t_R$ (min)	$^{2}t_R$ (s)	CAS	Formula
C8	8.86	0.36	111-65-9	C8H18
C9	13.06	0.40	111-84-2	C9H20
C10	17.96	0.52	124-18-5	C10H22
C11	23.06	0.57	1120-21-4	C11H24
C12	28.06	0.64	112-40-3	C12H26
C13	32.76	0.74	629-50-5	C13H28
C14	37.26	0.87	629-59-4	C14H30
C15	41.46	0.88	629-62-9	C15H32
C16	45.46	1.03	544-76-3	C16H34
C17	49.16	1.12	629-78-7	C17H36
C18	52.76	1.23	593-45-3	C18H38
C19	55.66	1.01	629-92-5	C19H40
C20	57.66	0.95	112-95-8	C20H42
C21	59.56	1.02	629-94-7	C21H44

Figure S3. Retention time vs n-alkanes series (Cn) plot for the calculation of the retention index by Van den Dol and Kratz equation

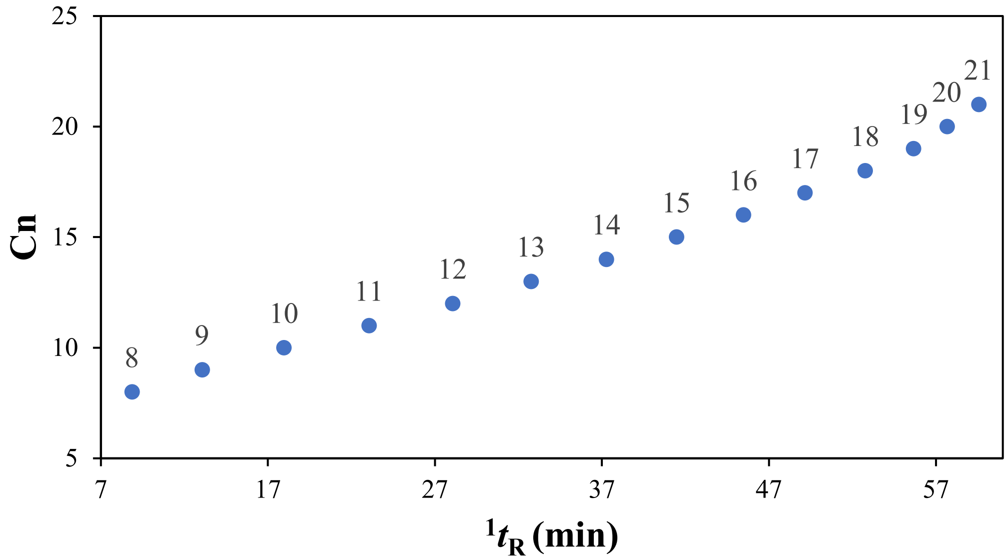

Figure S4. GC×GC–MS chromatogram for the HS–SPME of the series of n-alkanes (C8-C15)

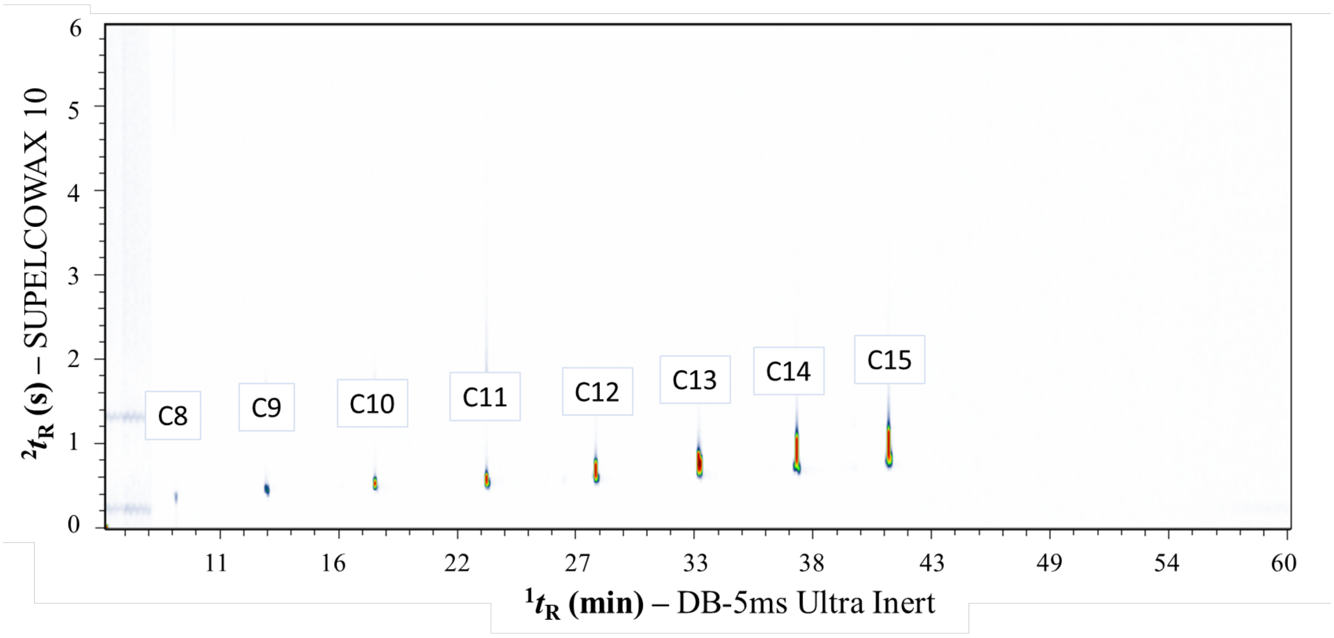

Figure S5. GC×GC–MS chromatogram for the HS–SPME of the series of n-alkanes (C16-C21)

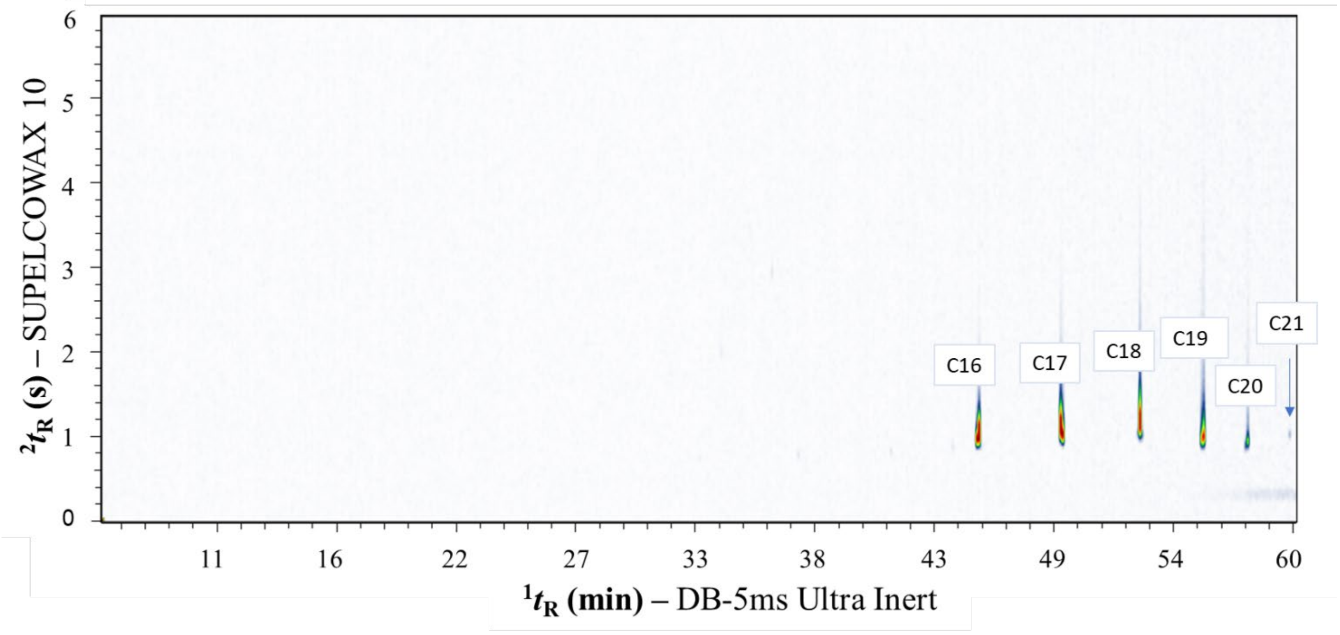

A comparison of the GC–MS and GC×GC–MS for the hop samples

Figure S6. Comparison of chromatograms obtained for the Enigma (ENIG) hop by HS–SPME using (A) GC–MS and (B) GC×GC–MS followed by the identification of selected peaks (C).

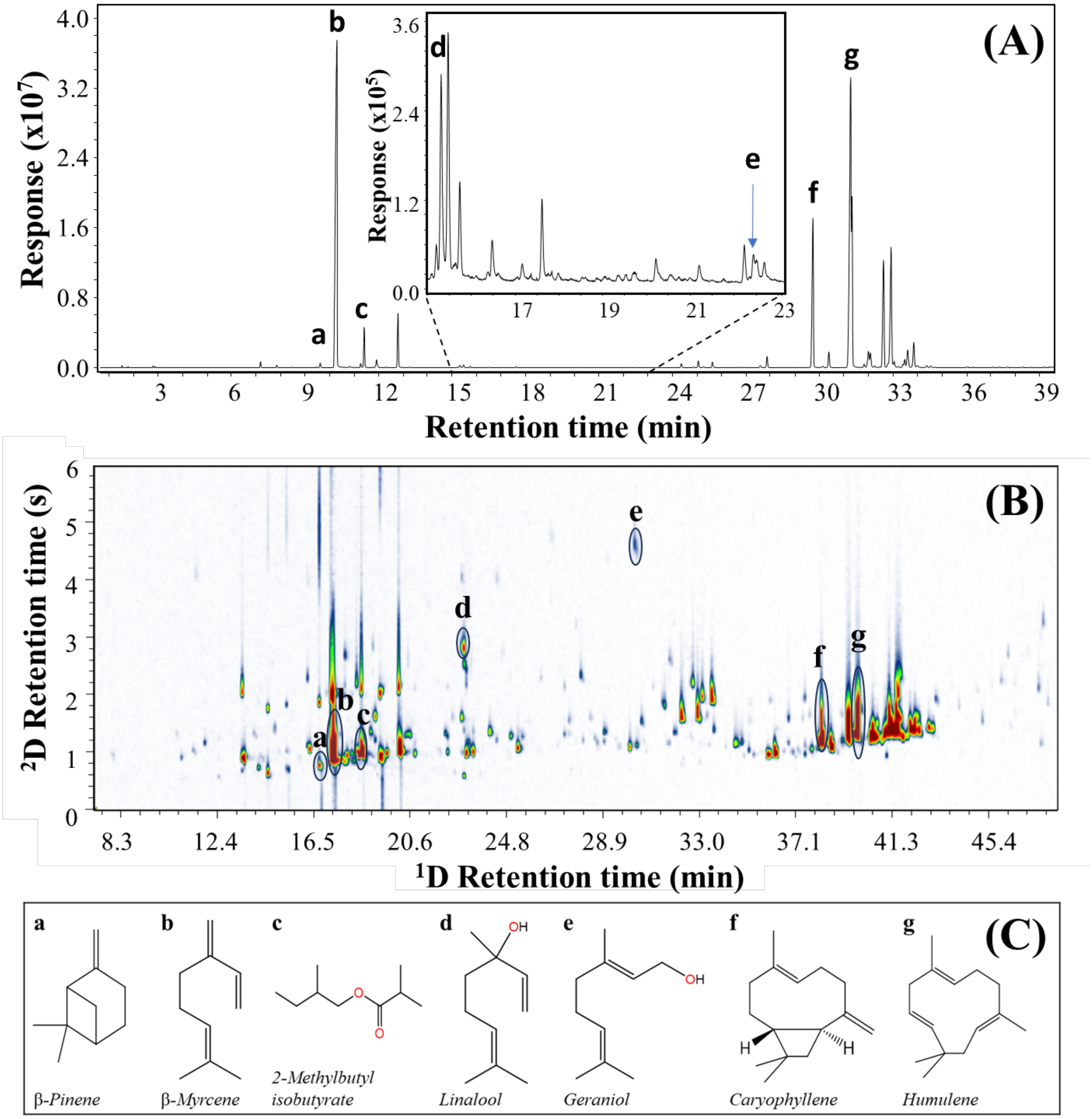

Figure S7. Comparison of chromatograms obtained for the Zappa (ZAPP) hop by HS-SPME using (A) GC–MS and (B) GC×GC–MS followed by the (C) identification of selected peaks.

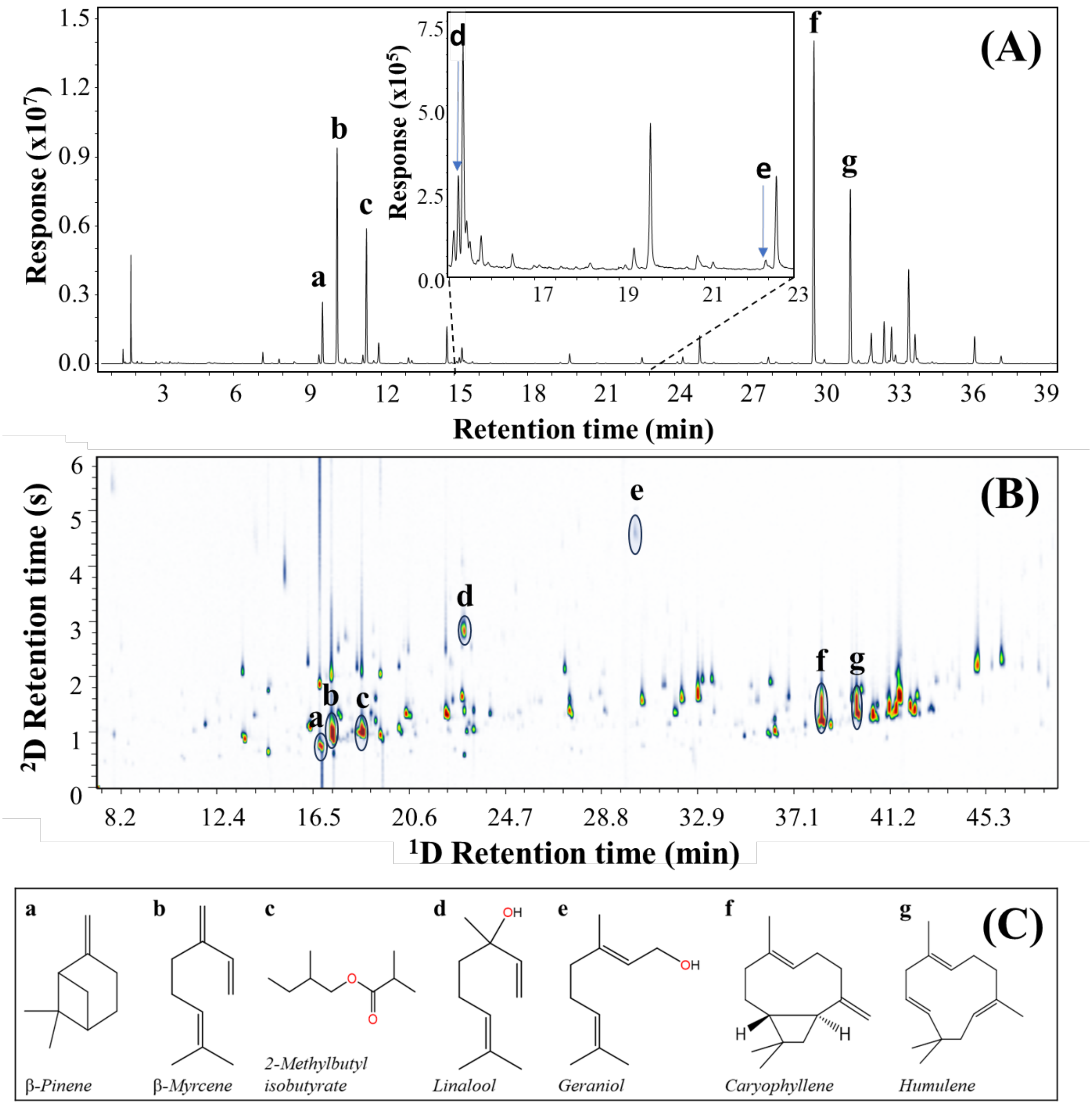

Table S4. Information in the hop composition profile using HS-SPME-GC–MS

No.	t_R (min)	Compound*	CAS	Formula	Lit. RI	Exp. RI	Relative GC chromatographic area (%) (Mean values ± SD for n=3)		
							CASC	ENIG	ZAPP
1	5.2	Propyl isobutyrate	644-49-5	C7H14O2	842±6	851	ND	0.0033 ± 0.0006	0.1461 ± 0.0062
2	6.0	2-Methylbutyl acetate	624-41-9	C7H14O2	880±3	876	ND	0.0046 ± 0.0009	0.0498 ± 0.0053
3	7.1	Isobutyl isobutyrate	97-85-8	C8H16O2	910±4	913	0.0214 ± 0.0018	ND	0.5585 ± 0.0317
4	7.8	α-Thujene	2867-05-2	C10H16	929±2	929	0.2195 ± 0.0085	ND	0.2758 ± 0.0170
5	7.8	α-Pinene	80-56-8	C10H16	937±3	930	0.2727 ± 0.0149	0.1174 ± 0.0222	ND
6	8.4	4,4-Dimethyl-2-buten-4-olide	20019-64-1	C6H8O2	952±5	944	0.1859 ± 0.0015	ND	0.1892 ± 0.0138
7	8.5	Camphene	79-92-5	C10H16	952±2	945	ND	0.0339 ± 0.0069	ND
8	9.5	2-Methylbutyl propionate	2438-20-2	C8H16O2	970±4	970	ND	0.0427 ± 0.0100	0.5077 ± 0.0256
9	9.6	β-Pinene	127-91-4	C10H16	979±2	974	0.4879 ± 0.0344	0.2303 ± 0.0290	3.4566 ± 0.2572
10	10.1	6-Methyl-5-heptene-2-one	110-93-0	C8H14O	986±2	985	0.2180 ± 0.0152	ND	ND
11	10.3	β-Myrcene	123-35-3	C10H16	991±2	991	4.2778 ± 0.3663	29.5075 ± 2.5200	13.1833 ± 1.3945
12	10.8	Isobutyl 2-methylbutanoate	2445-67-2	C9H18O2	1004±4	1003	ND	0.0603 ± 0.0059	0.4956 ± 0.0592
13	11.3	Isoamyl isobutanoate	2050-01-3	C9H18O2	1015±3	1012	ND	0.1858 ± 0.0172	ND
14	11.4	2-Methylbutyl isobutyrate	2445-69-4	C9H18O2	1016±2	1016	0.4811 ± 0.0333	2.0756 ± 0.1742	7.9836 ± 0.6122
15	11.7	p-Cymene	99-87-6	C10H14O	1025±2	1022	0.4564 ± 0.0451	0.0112 ± 0.0049	0.2097 ± 0.0302
16	11.9	D-Limonene	5989-27-5	C10H16	1030±2	1026	1.5083 ± 0.1623	0.5335 ± 0.0425	1.3000 ± 0.1141
17	12.3	β-Ocimene	13877-91-3	C10H16	1037±7	1035	ND	0.0404 ± 0.0058	ND
18	12.8	trans-β-Ocimene	3779-61-1	C10H16	1049±2	1045	ND	2.8898 ± 0.2404	0.0819 ± 0.0039
19	13.1	Prenyl isobutyrate	76649-23-5	C9H16O2	1052±1	1052	ND	ND	0.3941 ± 0.0150
20	13.3	γ-Terpinene	99-85-4	C10H16	1060±3	1057	0.0208 ± 0.0040	ND	ND
21	13.9	*cis*-Linalool oxide	5989-33-3	C10H18O2	1074±4	1069	0.0507 ± 0.0077	ND	0.0257 ± 0.0026
22	14.7	Methyl 6-methyl heptanoate	2519-37-1	C9H18O2	NA	1086	ND	ND	2.1707 ± 0.0722
23	15.0	2-Nonanone	821-55-6	C9H18O	1092±2	1092	0.0293 ± 0.0037	ND	ND
24	15.1	Hop ether	344294-72-0	C10H16O	NA	1095	0.1956 ± 0.0419	0.0021 ± 0.0007	0.1472 ± 0.0358
25	15.2	Perillen	539-52-6	C10H14O	1101±2	1097	1.1136 ± 0.1940	0.0174 ± 0.0064	0.3953 ± 0.0452
26	15.3	Linalool	78-70-6	C10H18O	1099±2	1099	1.2597 ± 0.2218	0.1266 ± 0.0350	1.0377 ± 0.0886
27	15.5	2-Methylbutyl 2-methylbutanoate	2445-78-5	C10H20O2	1105±2	1103	ND	0.1464 ± 0.0235	0.1099 ± 0.0132
28	15.7	2-Methylbutyl isovalerate	2445-77-4	C10H20O3	1107±2	1108	0.1321 ± 0.0258	0.0471 ± 0.0084	0.1106 ± 0.0082

29	16.5	Methyl octanoate	111-11-5	C9H18O2	1126±2	1124	ND			0.0215	±	0.0163	0.0677	±	0.0061
30	17.2	(4E,6E)-Allocimene	3016-19-1	C10H16	1144±1	1138	ND			0.0106	±	0.0022	ND		
31	17.6	Hexyl isobutyrate	2349-07-7	C10H20O2	1150±2	1148	ND			0.0448	±	0.0123	ND		
32	19.7	Methyl 6-methyloctanoate	5129-62-4	C10H20O2	1193±5	1192	ND			ND			0.7060	±	0.0632
33	21.2	Methyl nonanoate	1731-84-6	C10H20O2	1225±2	1223	ND			0.0108	±	0.0030	ND		
34	22.2	HeptyI isobutyrate	2349-13-5	C11H22O2	1247±1	1246	ND			0.0190	±	0.0048	ND		
35	22.4	Geraniol	106-24-1	C10H18O	1255±3	1250	1.0079	±	0.0040	0.0224	±	0.0065	0.0516	±	0.0023
36	23.5	(Z)-Undec-6-en-2-one	107853-70-3	C11H20O	1274±NA	1274	0.2124	±	0.0244	ND			0.0634	±	0.0136
37	24.1	Methyl 8-methylnonanoate	5129-54-4	C11H22O2	1277±NA	1287	ND			ND			0.1505	±	0.0252
38	24.3	2-Undecanone	112-12-9	C11H22O	1294±2	1292	0.6527	±	0.0519	0.1883	±	0.0511	0.4179	±	0.0661
39	24.8	2-Undecanol	1653-30-1	C11H24O	1307±4	1302	ND			0.0395	±	0.0065	ND		
40	25.0	Methyl (Z)-4-decenoate	1191-02-2	C11H20O2	NA	1307	ND			0.3158	±	0.0763	1.6317	±	0.2994
41	25.6	*trans*-Geranic acid methyl ester	1189-09-9	C11H18O2	1324±2	1320	0.4456	±	0.0005	0.2622	±	0.0638	0.1026	±	0.0145
42	26.6	α-Cubebene	17699-14-8	C15H24	1351±2	1343	0.0306	±	0.0033	ND			0.0534	±	0.0043
43	26.7	n-Octyl Isobutyrate	109-15-9	C12H24O2	1346±3	1344	ND			0.0287	±	0.0074	ND		
44	27.3	2-Methyl-1-undecanal	110-41-8	C12H24O	1365±2	1358	0.0620	±	0.0098	ND			ND		
45	27.6	Ylangene	14912-44-8	C15H24	1372±2	1364	0.5011	±	0.0568	0.1145	±	0.0339	0.1948	±	0.0296
46	27.9	Copaene	3856-25-5	C15H25	1376±2	1371	1.8634	±	0.2077	0.5691	±	0.1031	0.5130	±	0.0783
47	28.5	(+)-Sativene	3650-28-0	C15H24	1396±0	1385	0.0429	±	0.0025	0.0113	±	0.0031	ND		
48	28.8	2-Dodecanone	6175-49-1	C12H24O	1396±9	1393	ND			ND			0.1124	±	0.0201
49	29.1	Isocaryophyllene	118-65-0	C15H24	1406±3	1398	ND			ND			0.0504	±	0.0095
50	29.5	cis-α-Bergamotene	18252-46-5	C15H24	1415±3	1409	ND			0.0187	±	0.0060	ND		
51	29.7	Caryophyllene	87-44-5	C15H24	1419±3	1414	12.9262	±	0.4946	9.4133	±	0.2803	23.6843	±	1.2782
52	30.1	β-Copaene	18252-44-3	C15H24	1432±3	1424	0.3567	±	0.0608	0.0667	±	0.0145	0.3057	±	0.0437
53	30.4	α-Bergamotene	13474-59-4	C15H24	1435±3	1429	1.9188	±	0.0244	0.8366	±	0.0969	ND		
54	31.3	Humulene	6753-98-6	C15H24	1454±3	1451	37.6843	±	0.8950	22.0169	±	1.0923	12.3631	±	1.1516
55	31.3	(E)-β-Famesene	18794-84-8	C15H24	1457±2	1452	ND			7.8769	±	0.2327	ND		
56	32.0	γ-Selinene	515-17-3	C15H24	1479±6	1468	ND			1.0421	±	0.0920	ND		
57	32.1	γ-Muurolene	30021-74-0	C15H24	1477±3	1470	3.6014	±	0.2510	0.8672	±	0.0684	2.6978	±	0.4025
58	32.6	β-Eudesmene	17066-67-0	C15H24	1486±3	1483	4.4862	±	0.1164	6.7028	±	0.3217	2.8692	±	0.4124
59	32.9	α-Selinene	473-13-2	C15H24	1494±3	1490	4.0265	±	0.2012	7.9672	±	0.3473	2.6219	±	0.3578
60	33.0	α-Muurolene	10208-80-7	C15H24	1499±3	1493	1.0388	±	0.2004	0.3349	±	0.0259	0.6550	±	0.0779

61	33.5	Methyl 3,6-dodecadienoate	16106-01-7	C13H22O2	NA	1504	ND			0.4518	±	0.0331	0.1824	±	0.0228
62	33.6	β-Bisabolene	495-61-4	C15H24	1509±3	1506	0.1976	±	0.1127	ND			ND		
63	33.6	γ-Cadinene	39029-41-9	C15H24	1513±2	1507	2.6860	±	0.2529	1.2033	±	0.0577	7.2685	±	0.5720
64	33.8	δ-Cadinene	483-76-1	C15H24	1524±2	1513	2.8384	±	0.1885	1.5243	±	0.0913	1.9931	±	0.2319
65	33.9	Calamenene	483-77-2	C15H22	1523±5	1516	0.9033	±	0.0064	ND			0.3754	±	0.0489
66	34.0	Zonarene	41929-05-9	C15H24	1527±N/A	1517	ND			0.1664	±	0.0134	ND		
67	34.4	Cadine-1,4-diene	16728-99-7	C15H24	1533±4	1527	0.0326	±	0.0003	0.1081	±	0.0199	0.0419	±	0.0068
68	34.6	α-Cadinene	24406-05-1	C15H24	1538±1	1531	0.3505	±	0.0123	0.0923	±	0.0152	0.1483	±	0.0282
69	34.7	α-Calacorene	21391-99-1	C15H20	1542±3	1535	0.2053	±	0.0014	ND			0.0583	±	0.0110
70	36.1	(Z)-Tetradec-6-en-2-one	NA	C14H26O	1570±N/A	1569	0.1466	±	0.0121	ND			ND		
71	36.3	Caryophyllene oxide	1139-30-6	C15H24O	1581±2	1575	0.5825	±	0.0260	ND			2.1205	±	0.1589
72	37.3	Humulene epoxide I	19888-33-6	C15H24O	1604±3	1602	0.3161	±	0.0358	0.0102	±	0.0011	0.0498	±	0.0056
73	37.4	Humulene epoxide II	19888-34-7	C15H24O	1606±2	1602	2.3933	±	0.0451	ND			0.6809	±	0.0629

Abbreviations: t_R – Retention time RI – Retention index; CASC –Cascade; ENIG –Enigma; ZAPP – Zappa; NA – Not applicable or not found; ND – Not detected.
* Tentative identification
[a] Lit. RI, literature retention indexes for the compounds on a semi-standard non-polar column, 5%-phenyl using NIST 11 library,
[b] Exp. RI, experimental retention index calculated by the Van den Dol and Kratz equation.

GC×GC–MS chromatograms

Figure S8. GC×GC-MS chromatogram for the HS-SPME of Azacca (AZAC) hop

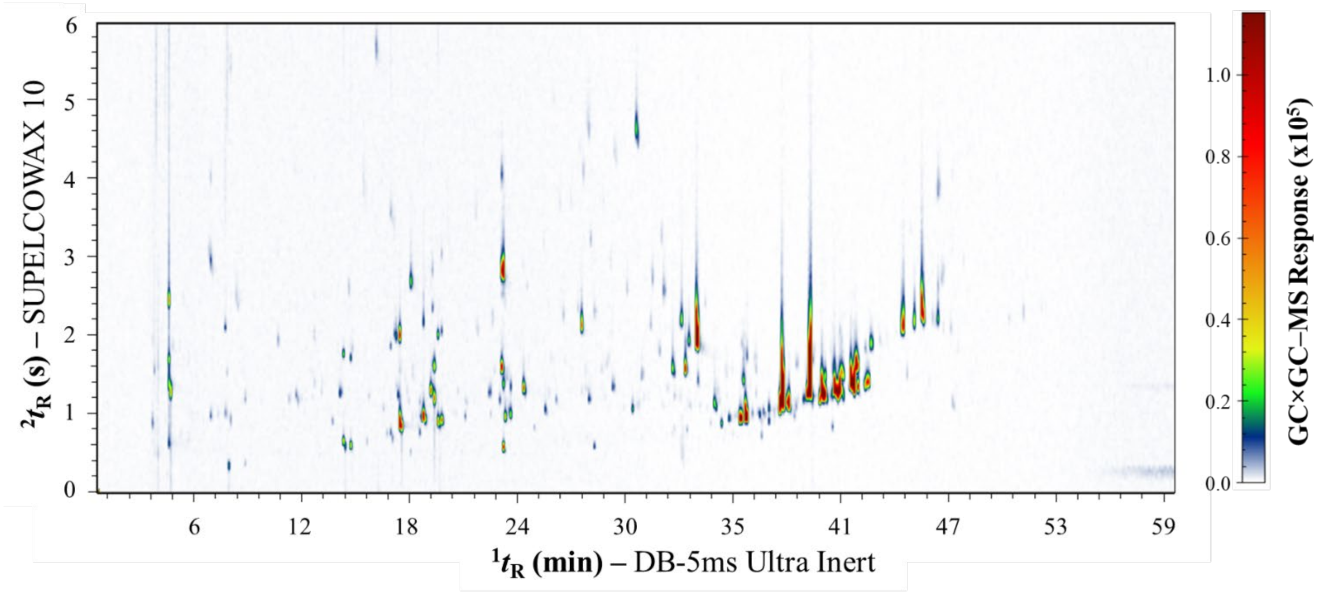

Figure S9. GC×GC-MS chromatogram for the HS-SPME of Loral (LORA) hop

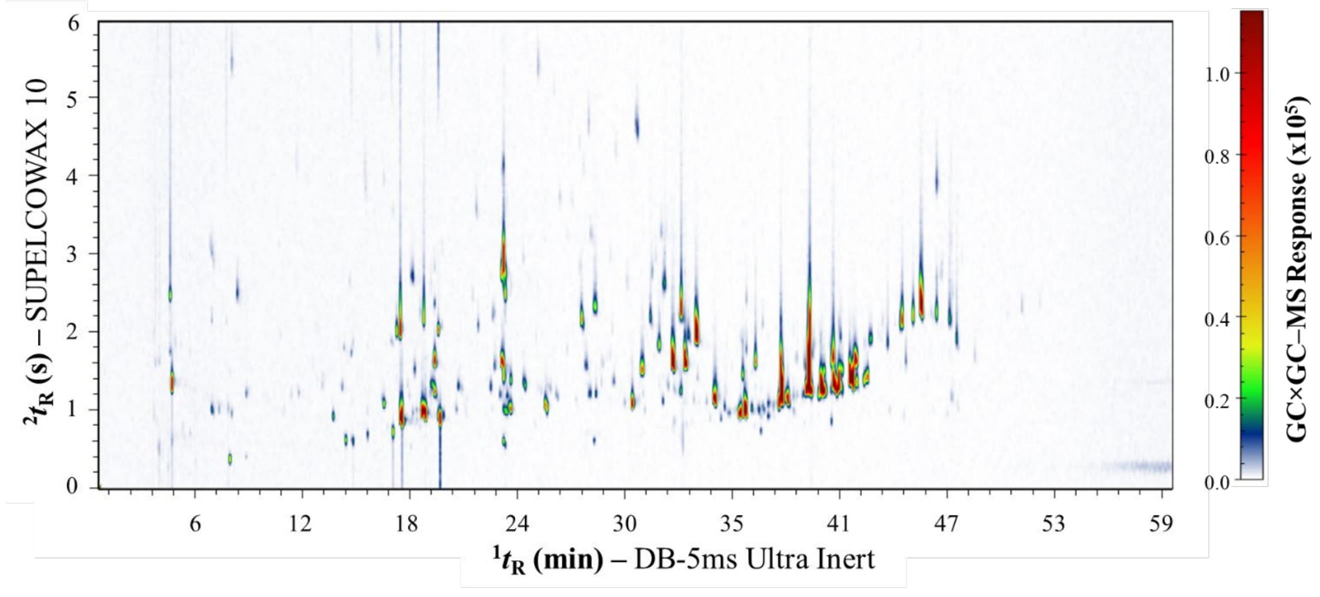

Supplement: Supplementary file 1 [file metabolites-14-00237-s001.zip › metabolites-2925015-supplementary.pdf]
